# Disruption of the gut microbiota in regulator of G protein signaling 14 knockout (RGS14 KO) mice alters the metabolome and reduces enhanced exercise capacity

**DOI:** 10.1007/s00421-026-06191-z

**Published:** 2026-03-14

**Authors:** Candace R. Longoria, Daniel D. DeSio, Marko Oydanich, Xiaoyang Su, Eric N. Chiles, Lee J. Kerkhof, Olufunmilola Ibironke, Max M. Häggblom, Nathan P. Wages, John J. Guers, Dorothy E. Vatner, Stephen F. Vatner, Sara C. Campbell

**Affiliations:** 1 Department of Kinesiology and Health, Rutgers, The State University of New Jersey, 70 Lipman Drive, New Brunswick, NJ 08901 USA; 2 Department of Cell Biology & Molecular Medicine, Rutgers University-New Jersey Medical School, Newark, NJ USA; 3 Cancer Institute of NJ, Metabolomics Shared Resource, Department of Medicine, Rutgers, The State University of New Jersey, New Brunswick, NJ USA; 4 Department of Marine and Coastal Sciences, Rutgers, The State University of New Jersey, New Brunswick, NJ USA; 5 Department of Biochemistry and Microbiology, Rutgers, The State University of New Jersey, New Brunswick, NJ USA; 6 Institute for Food, Nutrition, and Health, Center for Lipid Research, Rutgers, The State University of New Jersey, New Brunswick, NJ USA; 7 Center for Human Nutrition, Exercise, and Metabolism, Rutgers, The State University of New Jersey, Newark, NJ USA; 8 Center for Nutrition, Microbiome, and Health, Rutgers, The State University of New Jersey, New Brunswick, NJ USA; 9 Department of Rehabilitation and Movement Sciences, Rutgers University, Newark, NJ USA; 10 East Stroudsburg University, East Stroudsburg, PA USA

**Keywords:** Metabolites, brown adipose tissue, gut microbiota, antibiotics, skeletal muscle, exercise capacity

## Abstract

Regulator of G-protein Signaling 14 Knockout (RGS14 KO) mice exhibit enhanced exercise capacity and health span, however the contribution of the gut microbiota to this phenotype remains unclear. This study integrated long-read rRNA operon amplicon sequencing and metabolomics to first determine how microbial composition and tissue metabolite profiles differ between RGS14 KO and their wild-type littermates. Next, we investigated how administration of antibiotics to perturb the gut microbiota may affect the RGS14 KO phenotype. Prior to antibiotic treatment (ABX), RGS14 KO mice outperformed WT littermates in maximal running distance and work performed, accompanied by elevated skeletal muscle citrate synthase, complex IV activity, and nitric oxide production. One week of ABX significantly reduced exercise capacity in both genotypes and markedly suppressed mitochondrial activity in RGS14 KO skeletal muscle. Gut microbiota profiling revealed similar phylum-level structure between genotypes but distinct species- and strain-level signatures. Metabolomics of brown adipose tissue (BAT) and quadriceps identified genotype-specific metabolic programs that were disrupted by ABX, including pathways related to amino acid metabolism, nucleotide turnover, and mitochondrial energetics. Collectively, these findings demonstrate that RGS14 KO mice harbor unique microbial and metabolic networks that support enhanced thermogenesis and exercise performance, and that microbiota depletion eliminates these advantages. This work establishes a mechanistic foundation connecting the gut microbiota to BAT and skeletal muscle metabolism, highlighting potential microbiome-targeted strategies to improve metabolic health and physical performance.

**Supplementary Information:**

The online version contains supplementary material available at 10.1007/s00421-026-06191-z.

## Introduction

Mammalian adipose tissue exists in two primary forms, white (WAT) and brown (BAT); each with distinct roles in metabolic health and longevity. These adipose tissues are distinct from each other in anatomical location, morphology, gene expression patterns, biochemical features, and physiological functions (Cinti 2001; Park et al. 2014; Rosell et al. 2014; Giralt and Villarroya 2013). BAT, which possesses numerous mitochondria and multilocular lipid droplets, and is highly metabolically active due to its non-shivering thermogenic activity compared to WAT, which contains single lipid droplet that stores triglycerides with relatively fewer mitochondria (Rosell et al. 2014; Pilkington et al. 2021). Beige adipocytes are found within WAT and can transition through the process of ‘beiging’ or ‘browning’ between a WAT phenotype to take on more BAT-like activity following various stimuli such as cold exposure and exercise (Pilkington et al. 2021; Machado et al. 2022; Ghesmati et al. 2024). Beyond its thermogenic role, BAT has been acknowledged as a secretory organ capable of releasing molecules that act in paracrine or autocrine fashions (Cannon and Nedergaard 2004; Villarroya et al. 2017), including peptides, metabolites, and microRNAs (Yang and Stanford 2022), which communicate with other host tissues and organs.

The gut microbiota is an important modulator of host metabolic homeostasis and energy balance in BAT (Backhed et al. 2004; Turnbaugh et al. 2008; Tremaroli and Backhed 2012; Mestdagh et al. 2012; Suarez-Zamorano et al. 2015). BAT of germ-free (GF) mice have higher levels of myo-inositol, D-3-hydroxybutyrate, and glutamate and reduced levels of lactate compared to conventionalized GF mice (Mestdagh et al. 2012). In addition, both GF and antibiotic treated (ABX) mice displayed active browning of WAT, and ABX reduced WAT lipid droplet size (Suarez-Zamorano et al. 2015). Recent studies have explored connections between the gut microbiota and adipose tissue, including the browning of WAT, BAT thermogenic programming, and the effects of fasting, caloric restriction, cold stress, and metabolic endotoxemia (Rothwell and Stock 1979; Feldmann et al. 2009; Tomilov et al. 2014; Cohen et al. 2014; Himms-Hagen 1984; Ouellet et al. 2012). Mechanistic links between the gut microbiota and BAT remain poorly defined, and prior reviews of BAT biology have largely overlooked enteric influences (Ahmad et al. 2021). While studies have used GF mice and ABX to study aspects of interaction between BAT and the gut microbiota, including metabolism, thermogenesis, uncoupling protein 1 activity, and the beiging of WAT (Mestdagh et al. 2012; Suarez-Zamorano et al. 2015; Li et al. 2019), there is an absence of research investigating the mechanistic link between them.

Given the influence of the gut microbiota on metabolism, its role in exercise physiology and mitochondrial function warrants attention. Hsu et al. showed that both GF and gnotobiotic mice significantly underperformed mice that possessed a specific pathogen-free gut microbiota (Hsu et al. 2015). Further, a dose-dependent increase in time to exhaustion was observed in human subjects provided either a placebo, low, or high dose of probiotics (Huang et al. 2019). Additionally, increased gut microbiota richness has been linked to improved exercise performance (Hsu et al. 2015; Huang et al. 2019), supporting that gut microbiota abundance and composition play important roles in exercise-related outcomes. However, the mechanisms by which gut microbiota alterations influence exercise capacity remain unclear.

Exercise promotes mitochondrial biogenesis and increases both transcription and translation of mitochondrial proteins (Popov 2020). Alternatively, mitochondrial dysfunction has been strongly associated with chronic disease development, genetic mutations, and aging (Nunnari and Suomalainen 2012; Chan 2020; Bravo-San Pedro et al. 2017). BAT-derived lipids such as 12,13-dihydroxy-9Z-octadecenoic acid are secreted during exercise and increase mitochondrial respiration and fatty acid uptake in skeletal muscle (SKM) (Stanford et al. 2018), directly connecting BAT and SKM. Our prior work demonstrated that one week of ABX eliminates exercise adaptations, including improvements in exercise capacity, blood flow, and markers of mitochondrial biogenesis (Dowden et al. 2023). Additionally, our previous data showed that BAT from the Regulator of G Protein Signaling 14 Knockout (RGS14 KO) mouse, a model of healthful aging and enhanced exercise capacity, contributes to its performance phenotype, and surgical removal of RGS14 KO BAT into its wild-type (WT) littermate confers this fitness ability (Vatner et al. 2023). These findings reinforce the role of mitochondria in exercise physiology, but the ability of the gut microbiota to influence metabolically active and mitochondria-dense tissues such as BAT and SKM remains unexplored.

To address this gap, we employed an integrated metabolomics and long-read rRNA amplicon sequencing approach using the Oxford Nanopore Technology MinION to obtain metabolite profiles and bacterial species/strain-specific data using the RGS14 KO mouse model of enhanced BAT and exercise capacity. This approach enables rapid sequencing and identification of bacterial rRNA operons with species-level detection (Kerkhof et al. 2017; Ibironke et al. 2020). We also employed liquid chromatography coupled with mass spectrometry to identify metabolites from RGS14 KO and WT littermate BAT and SKM. We hypothesized that RGS14 KO mice harbor a distinct gut microbiota and tissue-specific metabolite profile compared to WT littermates, and these differences play a role in promoting BAT function and exercise capacity. We also hypothesized that ablation of the gut microbiota using an antibiotic cocktail would disrupt these microbial and metabolic networks. Our findings indicate that while both RGS14 KO and WT littermates share a similar phylum-level gut microbiota composition, they differ significantly at the species and strain level. Metabolomics analysis revealed significantly different metabolites when compared between RGS14 KO and WT littermates before and after ABX. These results begin to bridge the gap linking gut microbiota to BAT and SKM metabolomes, highlighting microbial and metabolic pathways that may underlie enhanced thermogenesis and exercise capacity. Ultimately, this work lays the foundation for microbiome-targeted strategies to improve metabolic health and performance.

## Materials and methods

### Study approval

All animals received humane care in compliance with the institution’s guidelines, as outlined in the Guide for the Care and Use of Laboratory Animals published by the National Institutes of Health. Experiments were completed at Rutgers University and approved by the Rutgers University Institutional Animal Care and Use Committee.

### Generation of RGS14 KO mice

C57BL/6J background mice with systemic RGS14 gene deficiency were developed as previously described (Lee et al. 2010). RGS14 KO mice (RGS14tm1-lex) were generated in the Transgenic Core Facility at Rutgers University New Jersey Medical School, through the National Institutes of Health-sponsored Mutant Mouse Regional Resource Center. Embryos were implanted into C57BL/6 females, and the founder mice were crossed with C57BL/6J to establish the RGS14 KO progeny. All mice were from F1 heterozygote crosses. Genotypes were determined using PCR of genomic DNA from mouse tails. After weaning (21 days), mice were placed in separate cages (4–5 mice per cage) in the same animal facility.

### Animal experimental procedures

Three- to six-month-old male RGS14 KO and their matched WT littermates were bred and housed at Rutgers University New Jersey Medical School. Mice were provided with the same standard chow (PicoLab Rodent Diet 20) and had ad libitum access to food and water for the length of the study in accordance with the Guide for the Care and Use of Laboratory Animals (National Research Council, Eighth Edition).

*Antibiotic Treatment*: The antibiotic cocktail used to eliminate the gut microbes consisted of 0.3 mg/ml each of ampicillin (Sigma-Aldrich A1593), neomycin (Sigma-Aldrich 1458009), metronidazole (Sigma-Aldrich, M1850000) and vancomycin (Sigma-Aldrich, M1709007) in the drinking water at an average rate of consumption of 3–5 ml/day for one week. Antibiotic-treated water was continuously available and was replaced every 2–3 days as previously described (Dowden et al. 2023). This combination of antibiotics has been widely utilized in studies ranging from 7 days to over 3 weeks as a reliable method of gut microbiota reduction (Kennedy et al. 2018). Mice receiving ABX were sacrificed within 24 h of completing the one-week treatment.

*Maximal exercise testing and relative critical power*: Mice receiving no ABX (*n* = 10, RGS14 KO; *n* = 6, WT littermates) or ABX (*n* = 10, RGS14 KO; *n* = 6 WT, littermates) were assessed for maximal running distance using a graded exercise test on a mouse treadmill (Accuscan Instruments Inc. AN5817474) (Guers et al. 2017; Vatner et al. 2015) before and after antibiotic administration. All mice were subjected to a practice trial 3 days before the experiments to adapt to the treadmill testing environment. At the time of the experiment, mice were placed on a treadmill with a 10% grade, which remained consistent throughout the test. The treadmill began at 4 m/min and the speed was incrementally increased 2 m/min every 2 min until the mice reached exhaustion, which was defined as spending 10 s on the electric stimulus platform without attempting to reengage the treadmill belt. To assess relative critical power, we used the slope of the linear regression line for work vs. time to exhaustion, controlling for body weight. By expressing values relative to body weight, we derived an index that allows meaningful comparisons across animals of varying body mass. Total distance run in meters and total work performed in joules are shown as J/g mean ± standard deviation and were compared using a two-way ANOVA paired with a Tukey’s multiple comparison post-hoc test. Figures were generated using GraphPad Prism version 10.2.3 and *p* < 0.05 was considered statistically significant for all tests.

*Weakness phenotype*s: To assess the weakness phenotypes of RGS14 KO and WT littermate mice, we established quartiles derived from relative distance to exhaustion and normalized these to respective animal body weight. Animal values that fell within the first quartile were considered weak, while animal values that fell within the fourth quartile were considered non-weak.

### Animal body weights

Body weights of no ABX (*n* = 14, RGS14 KO and *n* = 10, WT) and ABX (*n* = 8 RGS14 KO and *n* = 6 WT) mice at sacrifice are shown as mean ± standard deviation. Statistical comparisons were performed using a two-way ANOVA paired with Tukey’s multiple comparison post-hoc test. Figures were generated using GraphPad Prism version 10.2.3 and *p* < 0.05 was considered statistically significant.

### Blood, tissue and fecal sample collection

Male mice provided no ABX or one week of an antibiotic cocktail as previously described were anesthetized with pentobarbital sodium. Tissue samples were collected and stored as described below.

*Blood and plasma collection*: Blood was collected via cardiac puncture using a 5mL syringe. Following collection, samples were transferred to an EDTA-coated blood collection tube, stored on ice, and then centrifuged (Beckman Coulter AllegraX-15R) at 4 °C for 10 min at 10,000 rpm. Plasma samples were aliquoted and stored at -80 °C for later analysis.

*BAT collection*: Interscapular BAT was collected as previously described (Vatner et al. 2018b). Briefly, mice were placed in the prone position, and their backs were shaved in preparation for a 2 cm mid-scapular transverse incisions. BAT was carefully removed from surrounding muscles through the skin incision. After excision from the animal, BAT was carefully dissected to separate the surrounding WAT. BAT was then washed in 1x PBS, snap frozen in liquid nitrogen, and stored at -80 °C. Interscapular BAT was selected for its translatability, as it is similar in anatomical location to human infants (interscapular region) and human adults (cervical and supraclavicular regions) (Sidossis and Kajimura 2015).

*Quadriceps muscle collection*: Mice were placed in the supine position and skin was removed from the leg. Once the quadriceps was identified, fascia was removed to expose the muscle. Forceps were used to separate the muscle from the femur and cuts were made at the quadriceps tendon and origin of the rectus femoris. The quadriceps muscle was washed with 1x PBS, snap frozen in liquid nitrogen, and stored at -80 °C.

*Fecal collection*: Fecal pellets were collected from the distal colon at sacrifice using sterile tweezers. Samples were immediately snap frozen in sterile 1.5 microcentrifuge tubes using liquid nitrogen and stored at -80 °C for later analysis.

### Biochemical assays

The quadriceps tissue was homogenized, and protein concentration was determined using a Pierce BCA protein assay kit (Thermo Fisher; 23225) prior to performing assays. Citrate synthase activity was measured using the citrate synthase activity kit (Cayman Chemicals; 701040). Complex IV activity was measured using the Complex IV rodent assay kit (Cayman Chemicals; 700990). Adenosine monophosphate kinase (AMPK) phosphorylation was measured using an ELISA (LSbio; LS-F36058O). Total nitric oxide (NO) was measured using the nitrate/nitrite fluorometric assay kit (Cayman Chemicals; 780051). Sirtuin-1 (SIRT1) activity was measured using the fluorometric SIRT1 activity assay kit (Abcam; ab156065). Sirtuin-3 (SIRT3) was measured using the fluorometric SIRT3 activity assay kit (Abcam; ab156067). All assays were performed as recommended by the manufacturers. Data are shown as mean ± standard deviation. Means were calculated and statistical differences were compared using two-way ANOVA with a Tukey’s post hoc test and figures generated using GraphPad Prism version 10.2.3 with a *p* < 0.05 considered statistically significant.

### Plasma analyte concentrations

A Luminex MagPix mouse cytokine/chemokine panel 1 (MCYTOMAG-70 K, Millipore Sigma) was used to analyze systemic immune/inflammation biomarkers and determine the influence of ABX. Data are shown as mean ± standard error of the mean. Means were calculated and statistical differences were compared using two-way ANOVA with a Tukey’s post hoc test and figures generated using GraphPad Prism version 10.2.3 with a *p* < 0.05 considered statistically significant.

### Metabolite extraction, analysis, and metabolomics

#### Homogenization of tissues for metabolomics

Keeping the tissue frozen, between 10 and 30 mg of tissue was weighed and added to a 2 mL microcentrifuge tube along with a 6.5 mm Yttria Stabilized Zirconia Grinding Media Ball. Microcentrifuge tubes were kept chilled on dry ice prior to sample homogenization. The samples were homogenized under liquid nitrogen flow using the Retsch CryoMill, which was set to 20 Hz and run in 2-minute cycles until the samples were of a fine powdery consistency.

#### Metabolite extraction from tissues

Metabolites were extracted using a protocol optimized for water soluble polar metabolite analysis using liquid chromatography coupled with mass spectrometry. The extraction buffer used was a (v/v/v) solution of 40:40:20 (methanol: acetonitrile: water) + 0.1 M formic acid stored at -20 °C prior to usage. Immediately preceding the metabolite extraction, an aliquot volume equivalent to 20x the sample weight was added to the microcentrifuge tube with the homogenized sample, vortexed for 10 s, and left to chill on crushed ice for 10 min. The samples were then centrifuged at 4 °C for 10 min at 16,000 x g (Thermo Scientific Pico 21 Microcentrifuge). The supernatant was transferred to a correspondingly labeled and chilled microcentrifuge tube, and the process was repeated once more. The total volume of supernatant was then centrifuged for an additional 10 min. After centrifugation, a final 500 µL aliquot of the homogenate was then pipetted to a second clean microcentrifuge tube, to which 44 µL of 15% (m/v) ammonium bicarbonate (NH_4_HCO_3_) was added to neutralize the acid in the buffer.

#### Ultra-High-performance liquid chromatography conditions

Hydrophilic interaction chromatography separation was performed on a Vanquish Horizon ultra-high-performance liquid chromatography system (Thermo Fisher Scientific, Waltham, MA) with XBridge BEH Amide column (150 mm × 2.1 mm, 2.5 μm particle size, Waters, Milford, MA) using a gradient of solvent A (95%:5% water: acetonitrile with 20 mM acetic acid, 40 mM ammonium hydroxide, pH 9.4), and solvent B (20%:80% water: acetonitrile with 20 mM acetic acid, 40 mM ammonium hydroxide, pH 9.4). The gradient was 0 min, 100% B; 3 min, 100% B; 3.2 min, 90% B; 6.2 min, 90% B; 6.5 min, 80% B; 10.5 min, 80% B; 10.7 min, 70% B; 13.5 min, 70% B; 13.7 min, 45% B; 16 min, 45% B; 16.5 min, 100% B and 22 min, 100% B (Su et al. 2020). The flow rate was 300 µL/min. Injection volume was 5 µL, the column temperature was 25 °C, and the autosampler temperature was set to 4 °C.

#### Full scan mass spectrometry

The full scan mass spectrometry analysis was performed on a Thermo Q Exactive PLUS with a HESI source which was set to a spray voltage of -2.7 kV under negative mode and 3.5 kV under positive mode. Sheath, auxiliary, and sweep gas flow rates of 40, 10, and 2 (arbitrary units), respectively, were used. The capillary temperature was set to 300 °C and auxiliary gas heater was set to 360 °C. The S-lens RF level was 45. The m/z scan range was set to 72 to 1000 m/z under both positive and negative ionization mode. The AGC target was set to 3 × 10^6^ and the maximum IT was 200 ms. The resolution was set to 70,000 (Su et al. 2020).

#### Metabolomics

Data were collected using the MAVEN software package with a mass accuracy window set to 5 ppm, and all statistical analyses of the data were subsequently performed in R (3.6.1) (Melamud et al. 2010). The metabolite annotations are based on accurate mass-to-charge ratio (m/z) and retention time. Boxplots and heatmaps were generated with the ggplot2 package in R to compare mean metabolite levels and metabolite fold changes between RGS14 KO and WT groups, respectively. Statistical analysis in the plots was generated via a groupwise Student’s t-test paired with the ggom_signif function, features found in the ggsignif package in R. For all Student’s t-tests, a *p* < 0.05 was considered statistically significant.

#### Metabolite pathway analysis

MetaboAnalyst 6.0 was used to analyze topological metabolite pathways in BAT and quadriceps tissue. Briefly, workflow consisted of the following: (1) Selection of pathway analysis within annotated features, (2) Input of compound names, (3) Successful compound hits by matching with Kyoto Encyclopedia of Genes and Genomes IDs, and (4) Selection of the ‘*Mus musculus’* Kyoto Encyclopedia of Genes and Genomes pathway library. A pathway impact *≥* 0.05 and a false discovery rate of p < 0.05 were required for a metabolic pathway to be considered significant. Statistical analyses were calculated by the MetaboAnalyst 6.0 online database (Chong et al. 2019).

### DNA extraction and amplification, and gut microbiome analysis

#### Bacterial DNA extractions and purification

Twenty-two fecal samples (*n* = 13 RGS14 KO, *n* = 9 WT) were collected from the distal colon, snap frozen in liquid nitrogen, and stored at − 80 °C. DNA was extracted using a cetyltrimethylammonium bromide phenol/chloroform extraction method with 5 rapid freeze/thaw cycles and ethanol precipitation as previously described (Dowden et al. 2020). The DNA extracts were further purified using Agencourt AMPure beads (Beckman Coulter, Brea, CA, USA) and stored at − 80 °C until PCR.

#### Ribosomal RNA (rRNA) operon amplifications

Near full-length bacterial rRNA operons were amplified via PCR with universal bacterial 16 S rRNA forward 27 F (5′- AGA GTT TGA TCC TGG CTC AG-3′) and 23 S rRNA reverse 2241R (5′ -ACC GCC CCA GTH AAA CT-3′) primers attached to Oxford Nanopore Technology barcode sequences for multiplex sequencing. DNA extracts (< 10 ng template DNA) were amplified using TaKaRa Taq DNA Polymerase (Takara Bio USA, Inc, CA, USA). A touchdown PCR program for the bacterial operons included an initial denaturation of 5 min at 95 °C; 2 cycles of 95 °C/20 seconds for denaturation, 68 °C/15 seconds for primer annealing, 72°/75 seconds for extension; then 2 cycles of 66 °C for primer annealing; 2 cycles of 64 °C for primer annealing; 2 cycles of 62 °C for primer annealing-all with denaturation/extension; followed by 22 cycles of denaturation, 60 °C/15 seconds for primer annealing, extension; and a final extension at 72 °C for 5 min. PCR amplification utilized 30 cycles in total and rRNA operon amplicons were visualized/quantified by agarose gel electrophoresis.

#### Library preparation and sequencing by MinION

MinION library construction utilized the 1D sequencing kit (SQKLSK109-Oxford Nanopore; Oxford, UK). All 22 barcoded amplicons (each sample having ~ 150 ng of DNA, 1,800 ng in total) were combined, end-repaired, and dA-tailed using the NEB Ultra II end-repair kit (New England Biolabs, Ipswich, MA, USA). Ligation of the Oxford Nanopore Technology adaptor utilized the Blunt/TA ligase master mix (NEB) with an addition of 1 µL of freshly prepared adenosine triphosphate solution (∼4 mg/mL) to facilitate ligation. All libraries were analyzed on R9.4 flow cells as previously described (Ibironke et al. 2020).

#### Microbiome classification

MinION fast5 files were base called using Guppy (4.2.2). Raw reads were separated by barcode, sized between 3700 and 5700 bp in length, and screened against an rRNA operon database to determine Best BLAST Hits using MegaBLAST settings for strain level resolution (Kerkhof et al. 2022). BLAST settings utilized a word size of 44, gap reward/penalty of 2; -3, gap open/extend penalty of 0; -4, an e-value of 10, and a max_target_seq of 1. This approach has been shown to provide strain level resolution for long-read MinION sequences with matches to the ribosomal RNA operon database with an alignment with > 1,000 bp and > 84% identity (Kerkhof et al. 2022).

#### Non-metric multidimensional scaling and diversity analysis

The vegan package (Oksanen 2019) was used in R (3.6.1) to compute a log transformation of the raw sequencing data from each sample (*n* = 22) and create a dissimilarity matrix using a Kulczynski distance. The number of dimensions (K = 2) were used for the analysis based on the non-metric multidimensional scaling stress plots and R^2^ values for model fit. The R^2^ value for non-metric fit of observed dissimilarities was ~ 0.99 for all data analyzed. Permutational Multivariate Analysis of Variance using the vegan ‘adonis’ code was performed to test for significance.

#### Differential abundance analysis

Classification results were copied into Excel and individual species counts were summarized via pivot tables. The DEseq2 package (Love et al. 2014) and ggplot2 package (Wickham 2016) were used to perform differential abundance analysis between RGS14 KO and WT data in R and used to generate volcano plots. Adjusted *p* < 0.05 and log_2_-fold-change > 2 were considered threshold values for differentially abundant bacteria. Other taxa exhibiting log_2_-fold-change > 2 in RGS14 KO mice but falling above the adjusted p value of < 0.05 are also presented.

## Results

### Animal biometrics

**Fig. 1:**
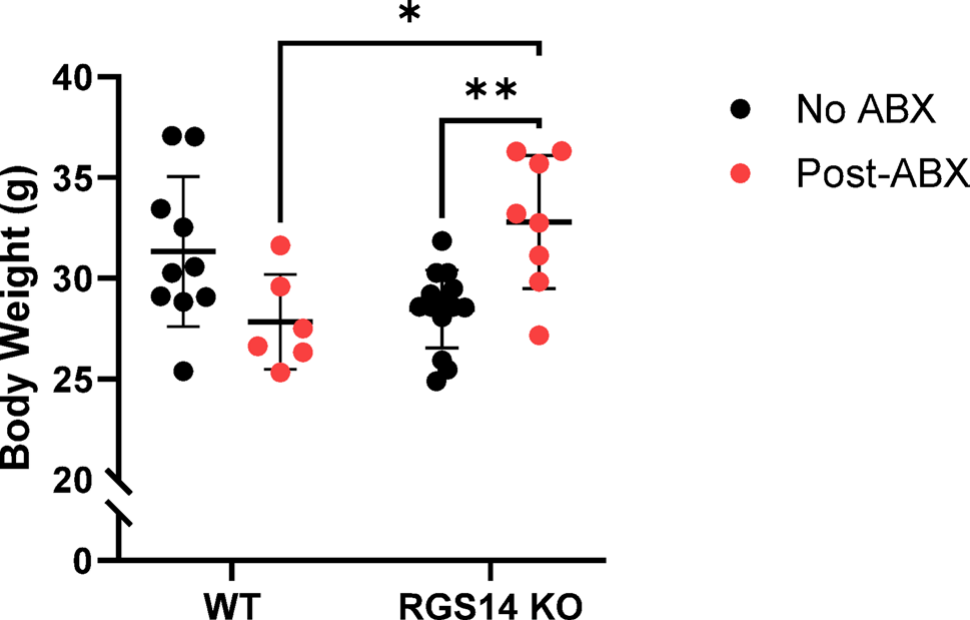
Animal body weights at sacrifice. RGS14 KO mice provided ABX weighed significantly more at sacrifice than RGS14 KO mice with no ABX or WT littermates administered ABX. Data shown as mean ± standard deviation. WT No ABX: *n* = 10, WT Post-ABX: *n* = 6; RGS14 KO No ABX: *n* = 14, RGS14 KO Post-ABX: *n* = 8. *: *p* < 0.05, **: *p* < 0.01

Following ABX, WT littermate mice weighed significantly less than RGS14 KO mice (27.8 ± 2.4 g vs. 32.8 ± 3.3 g; *p* = 0.014). RGS14 KO mice without ABX weighed significantly less than RGS14 KO mice provided ABX (28.5 ± 1.9 g vs. 32.8 ± 3.3 g; *p* = 0.009) (Fig. 1). No statistical differences were identified in WT littermate mice following ABX.

### Exercise performance

**Fig. 2:**
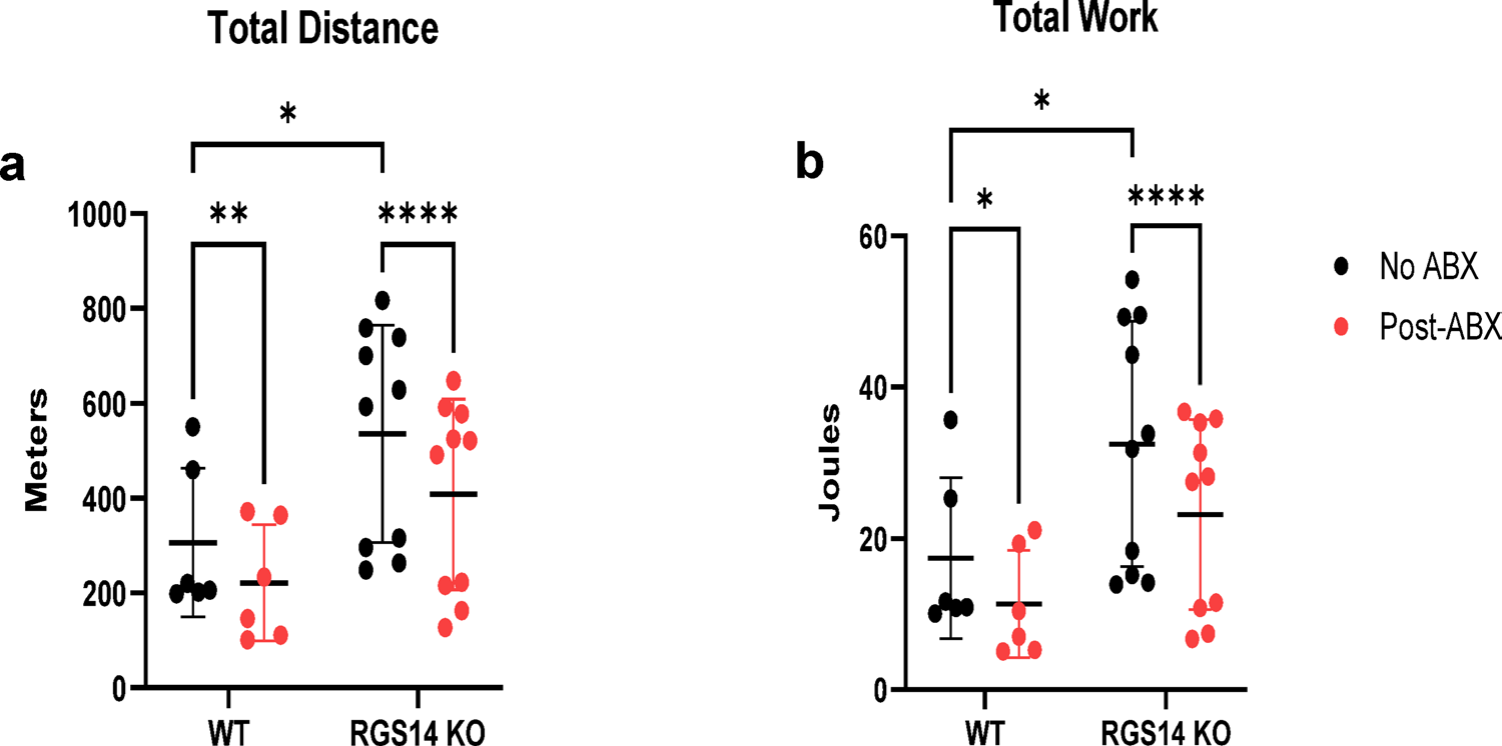
Maximal exercise testing. RGS14 KO mice significantly outperformed WT littermates prior to ABX administration in total distance run **(A)** and work performed **(B)**. Following ABX, both RGS14 KO and WT littermates demonstrated significantly reduced performance in both outcomes compared to respective genotypes provided no ABX. Data shown as mean ± standard deviation. WT No ABX: *n* = 6, WT Post-ABX: *n* = 6; RGS14 KO No ABX: *n* = 10, RGS14 KO Post-ABX: *n* = 10. *: *p* < 0.05, **:*p* < 0.01, ***: *p* < 0.001, ****: *p* < 0.0001

Prior to ABX, RGS14 KO mice significantly outperformed WT littermates in total distance run (536.1 ± 228.6 m vs. 306.4 ± 157.2 m; *p*=0.028) and total work performed (32.5 ± 16.2 J vs. 17.4 ± 10.7 J; *p*=0.031), which we have previously demonstrated ADDIN EN.CITE (Vatner et al. 2023). RGS14 KO mice demonstrated a significant reduction after ABX for both total distance run (536.1 ± 228.6 m vs. 408.7 ± 201.2 m; *p*<0.0001) and total work performed (32.5 ± 16.2 J vs. 23.2 ± 12.6 J; *p*<0.0001). This significant reduction was also identified in WT littermate mice following ABX for total distance run (306.4 ± 157.2 m vs. 221.5 ± 123.1 m; *p*=0.006) and total work performed (17.4 ± 10.7 J vs. 11.4 ± 7.1 J; *p*=0.012) (Fig. 2).

### Critical power outcomes and weakness phenotypes

Critical power, which is quantified as the relationship between the slope of work performed vs. time, represents the highest metabolic rate at which exercise can be theoretically sustained without a progressive, fatigue-driven decline in performance (Hill 1925; Poole et al. 2016). It demarcates the boundary between the heavy-intensity exercise domain, where physiological variables such as oxygen uptake and metabolite concentrations can stabilize, and the severe-intensity exercise domain, where homeostasis cannot be maintained and exhaustion becomes overwhelming (Chidnok et al. 2013; Muniz Pumares and Meyler 2025). The relative critical power index, which normalized critical power to individual body weight, revealed a significant genotype-dependent effect (Table 1). Specifically, WT littermate mice demonstrated significantly lower relative critical power when compared to RGS14 KO animals both before (13.3 vs. 19.3 J/g, *p* < 0.05) and after ABX (8.6 vs. 14.3 J/g, *p* < 0.05).

**Table 1:** Relative critical power by genotype. Critical power outcomes of RGS14 KO and WT littermate mice

Animal Genotype	Body weight (g)	CP before ABX	CP after ABX	Mean CP Difference	Percent Change
WT^‡^	25.3	17.2	13.1	4.1	23.9
WT^‡^	26.3	22.3	14.6	7.7	34.5
WT	29.6	8.6	5.2	3.4	39.6
WT	27.5	10.7	6.2	4.5	41.9
WT*	***26.6***	***10.1***	***7.7***	***2.3***	***23.2***
WT*	***31.6***	***11.2***	***4.6***	***6.6***	***58.8***
Average (Weak and Non-weak)	27.8	13.3	8.6	4.8	37.0
Non-weak only^‡^	29.1	13.8	5.9	29.2	19.7
Weak only*	25.8	6.2	4.5	41.0	10.7
***RGS14 KO*** ^***‡***^	***33.2***	***27.1***	***20.8***	***6.2***	***23.0***
***RGS14 KO*** ^***‡***^	***30.8***	***18.9***	***16.3***	***2.6***	***14.0***
RGS14 KO	27.2	9.5	5.7	3.7	39.4
RGS14 KO	31.2	12.7	8.7	4.0	31.8
RGS14 KO	35.7	25.8	20.8	5.0	19.5
RGS14 KO	36.3	27.4	18.6	8.8	32.0
RGS14 KO	36.3	31.6	21.3	10.3	32.7
RGS14 KO	33.7	19.6	17.6	2.0	10.0
RGS14 KO*	***32.8***	***10.5***	***5.6***	***5.0***	***47.1***
RGS14 KO*	***29.8***	***10.1***	***7.6***	***2.5***	***24.7***
Average (Weak and Non-weak)	32.7	19.3	14.3	5.0	27.4
Non-weak only^‡^	32.0	18.6	4.4	18.5	23.0
Weak only*	31.3	6.6	3.7	35.9	10.3

*CP* critical power

*: indicates weak animals that fall into the first quartile category,

^‡^: indicates non-weak animals that fall into the fourth quartile.

Following assessment of weakness phenotypes within both RGS14 KO and WT littermates, two animals from each genotype fell into the weak or non-weak categories. The grouping of weak or non-weak animals allows for normalization of performance relative to individual animal size and thus reducing possible confounding changes in body weight following one week of ABX. Stratification by phenotypic weakness revealed significant differences between weak and non-weak animals across genotypes. Interestingly, weak animals from both WT littermates and RGS14 KO animals exhibited remarkably similar critical power values before (6.2 vs. 6.6 J/g) and after ABX (4.5 vs. 3.7 J/g).

### Quadriceps ELISAs

**Fig. 3:**
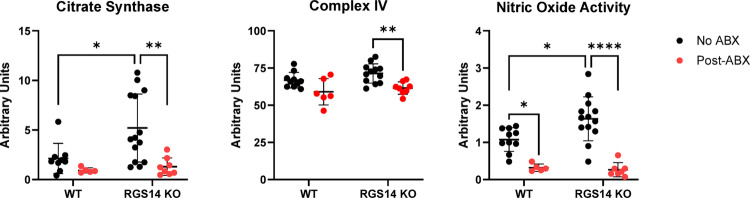
Markers of mitochondrial function and nitric oxide activity in skeletal muscle. **A** Citrate synthase activity was significantly higher in RGS14 KO mice compared to their WT littermates before ABX. Post-ABX, RGS14 KO mice presented with significantly decreased citrate synthase activity. **B** No significant differences were seen in complex IV activity before ABX; post-ABX, complex IV activity was significantly decreased in RGS14 KO mice. **C** Prior to ABX, nitric oxide activity was significantly higher in RGS14 KO mice compared to WT littermates. Post-ABX mice had significantly decreased nitric oxide activity compared to no ABX mice in both groups. WT no ABX: *n* = 9–10, WT post-ABX: *n* = 5–6, RGS14 no ABX: *n* = 12–14, RGS14 post-ABX: *n* = 8. Data shown as mean ± standard deviation. *:*p* < 0.05, **:*p* < 0.01, ***:*p* < 0.001, ****:*p* < 0.0001.

 To assess mitochondrial function, biogenesis, and NO activity in skeletal muscle without ABX and after ABX, we performed ELISAs to quantify citrate synthase, complex IV, NO, SIRT1, SIRT3, and AMPK using homogenized quadriceps tissue. Before ABX, RGS14 KO mice presented with a significantly higher baseline citrate synthase activity compared to WT littermates. Additionally, RGS14 KO mice post-ABX presented with a 74.9 ± 74.1% decrease in citrate synthase activity compared to pre-ABX RGS14 KO mice (Fig. 3a). Complex IV activity in RGS14 KO mice following ABX was significantly decreased when compared to RGS14 KO pre-ABX (Fig. 3b). Prior to ABX, RGS14 KO mice have a significantly higher level of NO activity compared to their WTL. After ABX, NO activity decreased in both WT littermate (70.2 ± 68.2%) and RGS14 KO (73.5 ± 46%) mice (Fig. 3c). Further, there was a significant effect of ABX for citrate synthase activity (*p* = 0.005), complex IV (*p* = 0.0002), NO activity (*p* < 0.0001), SIRT1 (*p* = 0.0274), and AMPK (*p* = 0.0100), but not SIRT3. No significant differences were identified between treatment groups for SIRT1, SIRT3, or AMPK (Suppl. Figure 1).

### Plasma analytes

A total of 32 analytes were analyzed from the plasma of WT and RGS14 KO mice before and after ABX (Table 2 and Suppl. Table 1). Significant increases in granulocyte-macrophage colony-stimulating factor (GM-CSF) and interleukin (IL)-12 p40 were detected in WT littermate mice post-ABX compared to no ABX; however, no significant differences in plasma analytes were identified between no ABX and post-ABX RGS14 KO animals. A significant effect of ABX and the plasma concentration increased for GM-CSF, IL-4, IL-5, IL-7, IL-12 p40, leukemia inhibitory factor, keratinocyte-derived chemokine, monocyte chemoattractant protein-1α, monocyte chemoattractant protein-1β, and monokine-induced by γ interferon after ABX. Additionally, a significant effect of ABX and a decrease in plasma concentration was measured for IL-1α, IL-2, and IL-10.

**Table 2:** MagPix plasma analyte values. Plasma analyte concentrations

Analyte	Group			
	WT	RGS14 KO	WT + ABX	RGS14 KO + ABX
G-CSF	15.3 ± 0.6	15.1 ± 0.3^a^	17.7 ± 1.5	15.5 ± 0.6
GM-CSF^*^	12.8 ± 0.4^a^	12.4 ± 0.3^a^	14.9 ± 1.1	13.3 ± 0.5
IL-1α^*^	120.3 ± 8.8	112.3 ± 6.2	85.6 ± 12.7	96.6 ± 12.0
IL-2^*^	107.6 ± 9.1	105.3 ± 6.5	82.9 ± 10.0	90.5 ± 12.2
IL-4^*^	12.7 ± 0.4	12.4 ± 0.3^a^	14.5 ± 1.0	13.4 ± 0.5
IL-5^*^	10.1 ± 0.2	10.2 ± 0.3	11.8 ± 1.1	10.8 ± 0.5
IL-7^*^	18.1 ± 0.5	17.8 ± 0.4^a^	20.8 ± 1.3	19.1 ± 0.8
IL-10^*^	71.7 ± 4.2	64.7 ± 2.2	60.9 ± 6.1	58.3 ± 5.2
IL-12 p40^*^	32.8 ± 1.7^a^	34.5 ± 1.3^a^	43.2 ± 4.4	36.6 ± 2.3
LIF^*^	27.4 ± 0.9	26.7 ± 0.7	30.1 ± 1.6	28.4 ± 1.2
KC^*^	21.0 ± 0.8	19.8 ± 0.6	23.6 ± 1.6	22.8 ± 1.5
MCP-1α^*^	21.9 ± 0.7	21.0 ± 0.5^a^	24.6 ± 1.5	22.5 ± 1.1
MCP-1β^*^	33.1 ± 1.3	31.4 ± 1.1	35.8 ± 2.3	34.5 ± 1.4
M-CSF^♯^	27.5 ± 3.0	22.6 ± 1.6	29.3 ± 3.0	23.3 ± 2.2
MIG^*^	20.9 ± 1.0	20.1 ± 0.6	23.4 ± 1.9	23.8 ± 1.7
RANTES^*^	62.9 ± 4.1	62.3 ± 4.0	47.6 ± 4.7	55.1 ± 7.5

Granulocyte colony-stimulating factor (G-CSF), granulocyte-macrophage colony-stimulating factor (GM-CSF), interleukin (IL), leukemia inhibitory factor (LIF), keratinocyte-derived chemokine (KC), monocyte chemoattractant protein-1α (MCP-1α), monocyte chemoattractant protein-1β (MCP-1β), macrophage colony-stimulating factor (M-CSF), monokine-induced by γ interferon (MIG), regulated on activation, normal T-cell expressed and secreted (RANTES). Data shown are mean analyte values (pg/µL) ± SEM

*: Significant effect of antibiotic treatment, *p* < 0.05. ^♯^: Significant effect of genotype, *p* < 0.05. ^a^: Significantly different vs. WT + ABX, *p* < 0.05

### Metabolite analysis in RGS14 KO and WT mice

All tissues were analyzed using liquid chromatography coupled with mass spectrometry under [M + H]^+^ and [M-H]^−^ ionization modes for 146 metabolites identified from the Rutgers Metabolomics Shared Resource in-house library.

### Metabolite comparison between RGS14 KO and WT before and after ABX

#### BAT

In untreated groups, eleven metabolites in BAT were significantly increased (*p* < 0.05) in RGS14 KO compared to WT littermate mice (Table 3). These included: the branched-chain amino acid (BCAA) isoleucine and metabolites involved in amino acid metabolism (2-hydroxyisocaproic acid and pipecolic acid); four metabolites related to energy metabolism (glucose-6-phosphate, malate, nicotinamide adenine dinucleotide phosphate, and UDP-D-glucose); the nucleosides guanosine and thymidine; the choline-derivative glycerophosphocholine, which is involved in cellular membrane structure and function; and glycyl-L-proline, a proline and glycine derivative. No BAT metabolites in WT littermates were significantly increased compared to RGS14 KO mice.

**Table 3:** Significantly Different Metabolites Detected in BAT. Significantly different BAT metabolites identified at baseline and following 1 week of antibiotic treatment

Metabolite	Significance	Charge		Increased Group	
Baseline					
G6P	*	+		KO	
GPC	*	+		KO	
Glycyl-L-Proline	**	+		KO	
Guanosine	*/**	-/+		KO	
Isoleucine	*	+		KO	
HICA	*	-		KO	
Malate	*	-		KO	
NADP+	*	-		KO	
Pipecolic Acid	*	-		KO	
Thymidine	*	-		KO	
UDP-D-Glucose	*	-		KO	
Post-ABX					
2-Hydroxyisovaleric acid	*	-		KO	
5-Methylcytosine	*	+		KO	
Betaine	*	+		WT	
Cystathionine	*	+		WT	
Cystine	*	-		KO	
Fructose	*	-		KO	
Glutathione	*	-/+		KO	
Guanine	*	-		KO	
HICA	*	-		KO	
L-Palmitoylcarnitine	*	+		WT	

Listed metabolites were identified as significantly different when comparing mean concentrations. HICA was significantly increased before and after ABX in RGS14 KO mice. Glucose-6-phosphate (G6P), glycerophosphocholine (GPC), 2-hydroxyisocaproic acid (HICA), nicotinamide adenine dinucleotide phosphate (NADP+), uridine diphosphate (UDP)-D-Glucose. +: metabolites identified with positive ionization mode; -: metabolites identified with negative ionization mode; -/+: metabolites identified with both positive and negative ionization modes. Charge significances with “/” denote significances in relation to positive or negative ionization modes; if there is no “/” in significance column, both – and + charges shared the same significance. ABX = antibiotic treatment, KO = RGS14 KO, WT = wild type

Significances: * = *p* < 0.05, ** = *p* < 0.01

Following 1 week of ABX, 7 metabolites in BAT were significantly increased (*p* < 0.05) in RGS14 KO compared to WT littermate mice. These included: the BCAA intermediates 2-hydroxyisovaleric acid and 2-hydroxyisocaproic acid; the amino acid derivative cystine; the nucleobase guanine and the methylated form of cytosine, 5-methylcytosine; fructose, which is related to energy metabolism, and the antioxidant glutathione. Additionally, 3 metabolites in RGS14 KO BAT were significantly decreased (*p* < 0.05) compared to their WT littermates. These included: betaine and cystathionine, both involved in homocysteine metabolism, and L-palmitoylcarnintine, a fatty acid metabolite involved in energy metabolism. Of the identified untreated and post-ABX metabolites in BAT, only 2-hydroxyisocaproic acid was identified as significantly increased both before and after ABX in RGS14KO mice (Table 3). Fold changes of metabolites identified as significantly different are shown as heat maps in Suppl. Figure 2).

#### Quadriceps

In untreated groups, a total of twenty metabolites in the quadriceps muscle were significantly different (*p* < 0.05) following metabolomics analysis; this included 15 metabolites that were significantly increased in RGS14 KO compared to WT littermate mice and 5 metabolites significantly decreased in RGS14 KO mice compared to WT littermates (Table 4). The 15 metabolites higher in the RGS14 KO animals included: the BCAAs leucine and isoleucine, the amino acid phenylalanine, and amino acid-associated metabolites acetyl glycine, acetyl methionine, and aspartyl phenylalanine. The B vitamin thiamine and cytidine 5’-diphosphocholine choline, an important metabolite in cellular structure and function were also significantly increased. Additionally, the nucleosides guanosine, inosine, and uridine, the purine metabolite hypoxanthine, and the energy metabolism metabolites guanosine monophosphate, ribose phosphate, and xylulose 5-phosphate were all significantly increased in the RGS14 KO mice compared to WT littermate mice. The 5 metabolites higher in WT littermates included: α-ketoglutarate, adenosine monophosphate (AMP), and nicotinamide adenine dinucleotide, which are involved in energy metabolism, the antioxidant glutathione, and the pyrimidine nucleotide intermediate N-carbamoyl-L-Aspartate.

**Table 4:** Significantly Different Metabolites Detected in Quadriceps. Significantly different quadriceps metabolites identified at baseline and following 1 week of antibiotic treatment

Metabolite	Significance	Charge		Increased Group	
Baseline					
α-Ketoglutarate	*	-		WT	
Acetyl glycine	*	-		KO	
Acetyl methionine	***/**	-/+		KO	
AMP	*/**	-/+		WT	
Aspartyl phenylalanine	***	-		KO	
CDP-Choline	*	-		KO	
Glutathione	*	-		WT	
GMP	*	-/+		KO	
Guanosine	*	+		KO	
Hypoxanthine	*	-		KO	
Inosine	***/**	-/+		KO	
Isoleucine	*	-/+		KO	
Leucine	**	-/+		KO	
NAD+	**	-/+		WT	
N-Carbamoyl-L-Aspartate	*	+		WT	
Phenylalanine	*	-/+		KO	
Ribose phosphate	**	-		KO	
Thiamine	*	+		KO	
Uridine	**	-		KO	
Xylulose-5-Phosphate	*	-		KO	
Post-ABX					
3-Phosphoserine	*	-		WT	
CDP-Ethanolamine	*	+		WT	
Glutamate	*	-/+		WT	
GPC	**/*	-/+		WT	
Hypotaurine	*	-/+		WT	
L-Arginino-Succinate	*	+		WT	
N-Acetyl-Glutamine	*	+		WT	
Pyridoxamine 5-Phosphate	*	+		WT	
Threonic acid	*	+		WT	
Thymidine	*	-		WT	
Thymine	*	-		WT	

Listed metabolites were identified as significantly different when comparing mean concentrations. Adenosine monophosphate (AMP), cytidine 5’-diphosphocholine (CDP)-choline/ethanolamine, guanosine monophosphate (GMP), glycerophosphocholine (GPC), nicotinamide adenine dinucleotide (NAD). +: metabolites identified with positive ionization mode; -: metabolites identified with negative ionization mode; -/+: metabolites identified with both positive and negative ionization modes. Charge significances with “/” denote significances in relation to positive or negative ionization modes; if there is no “/” in significance column, both – and + charges shared the same significance. ABX = antibiotic treatment, KO = RGS14 KO, WT = wild type

Significances: *:*p* < 0.05, **:*p* < 0.01, ***:*p* < 0.001

Following ABX, 11 metabolites were significantly reduced in the quadriceps of RGS14 KO mice compared to WT littermate mice. These included: 3-phosphoserine, a metabolite involved in serine metabolism, CDP-ethanolamine, an intermediate in phospholipid metabolism, the amino acid glutamate and the glutamine derivative N-acetyl-glutamine, the choline-derivative glycerophosphocholine, the taurine intermediate hypotaurine, the aspartic acid derivative L-Arginino-succinate, the vitamin B6 metabolite pyridoxamine 5-phosphate and the vitamin C metabolite threonic acid, the deoxynucleoside thymidine, and nucleobase thymine (Table 4). Fold changes of metabolites identified as significantly different are shown as heat maps in Suppl. Figure 3).

### Metabolic pathway analysis

Identified metabolites from UHPLC and full scan mass spectrometry were analyzed for topological metabolite pathways against the Kyoto Encyclopedia of Genes and Genomes PATHWAY database. This database is linked to the MetaboAnalyst 6.0 website and is a resource that includes a collection of pathway maps representing molecular interaction, reaction, and relation networks for metabolism activity (Kanehisa and Sato 2020). Following compound input to the MetaboAnalyst 6.0 website, no significant pathways were identified before or after ABX in either RGS14 KO or WT littermate mice in BAT. In the quadriceps, the purine metabolism pathway was identified as significant prior to ABX treatment (false discovery rate *p* < 0.05, pathway impact *≥* 0.05 (Suppl. Figure 4); as there were no metabolites identified as significantly increased in the quadriceps of RGS14 KO mice after ABX (Table 4), the purine metabolism pathway was no longer significant.

### Gut microbiome sequencing and community comparison

#### Ribosomal RNA operon sequencing

A total of ∼1.1 × 10^6^ raw amplicon reads were obtained, of which ~ 7 × 10^5^ reads passed Guppy basecalling (v4.2.2) and size selection (3.7–5.7 kb). Separation of these reads by barcode yielded ∼5 × 10^5^ sequences which were screened against a ribosomal RNA (rRNA) operon database with MegaBLAST (Kerkhof et al. 2022) (accession number provided for public access at acceptance). Overall, 1,615 bacterial species and 3,360 strains were detected in all fecal samples. The four dominant bacterial phyla representing ~ 98% of all reads for the RGS14 KO and WT littermate mice were *Bacteroidota* (formerly *Bacteroidetes*), *Bacillota* (formerly *Firmicutes*), *Pseudomonodota* (formerly *Proteobacteria*), and *Actinomycetota* (formerly *Actinomycetes*) (Suppl. Figure 5). The relative abundances of the 10 most abundant classes, orders, and families within RGS14 KO and WT littermates are presented in Suppl. Figures 6–8. The relative abundance of rRNA operon reads matching the top 50 bacterial strains are shown in Suppl. Figure 9 and include genera commonly found in the mouse gut microbiome.

#### Overall comparison of bacterial community between RGS14 KO and WT littermates

**Fig. 4:**
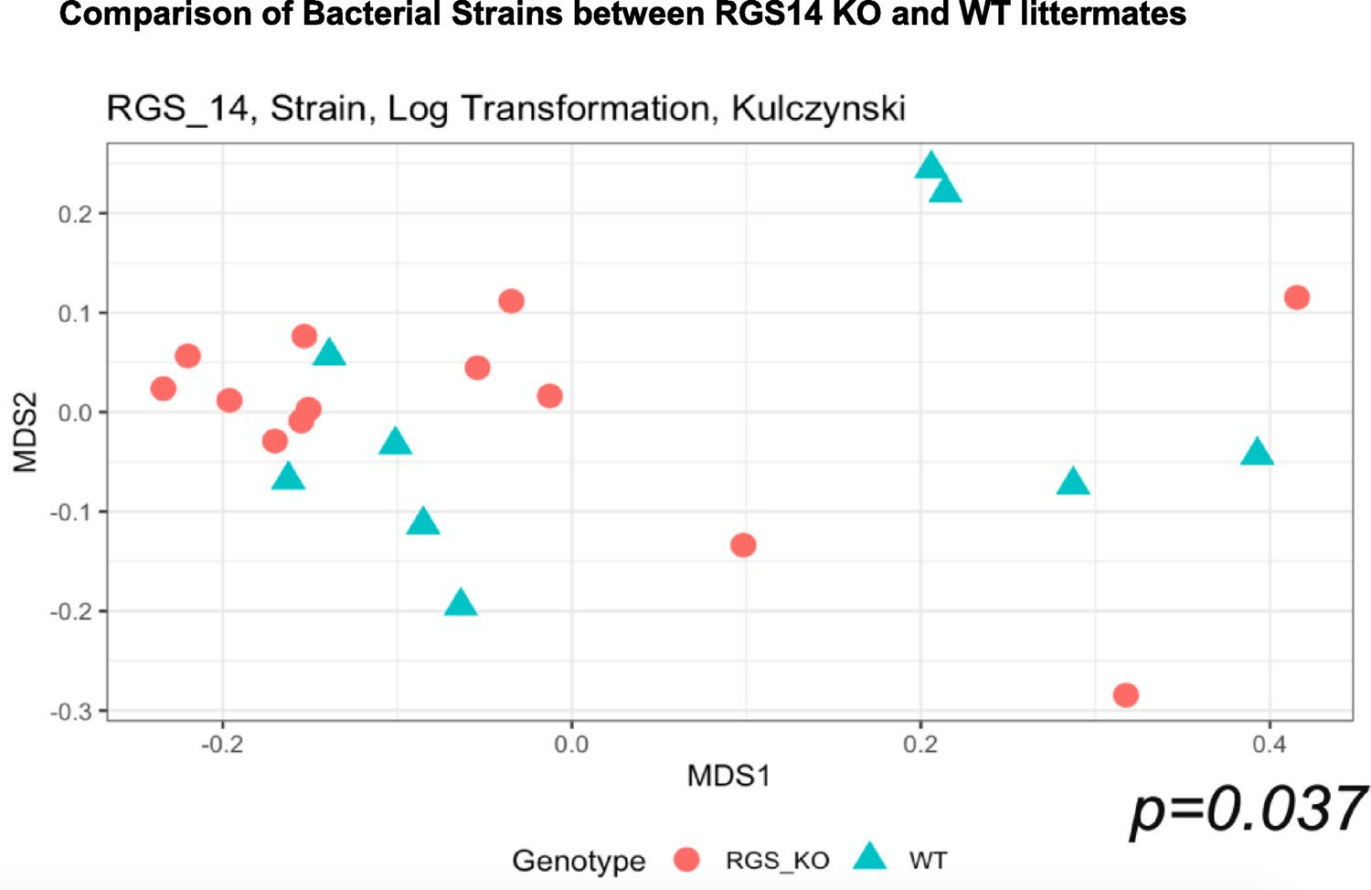
Non-metric multidimensional scaling ordination plots of Kulczynski distances based on bacterial strains of RGS14 KO and WT littermate mice. Each point represents an individual sample. Significance value refers to permutational multivariate analysis of variance test for distance differences between RGS14 KO and WT littermates

Similar composition of predominant bacteria was observed in the fecal microbiota of RGS14 KO and WT littermate mice (Suppl. Figure 9). To assess differences between the gut bacteria at finer taxonomic levels, the data was subjected to non-metric multidimensional scaling ordination analysis based on Kulczynski distance. Although there was little separation between RGS14 KO and WT littermate bacterial communities at higher taxonomic levels, a test of significance using a permutational multivariate analysis of variance paired with Kulczynski distance revealed significant differences between RGS14 KO and their WT littermates at the genus (*p* = 0.035) and species levels (*p* = 0.028) (Suppl. Figures 10 and 11), as well as the strain level (*p* = 0.037) (Fig. 4).

#### Comparison of differentially enriched taxa in RGS14 KO and WT littermates

**Fig. 5:**
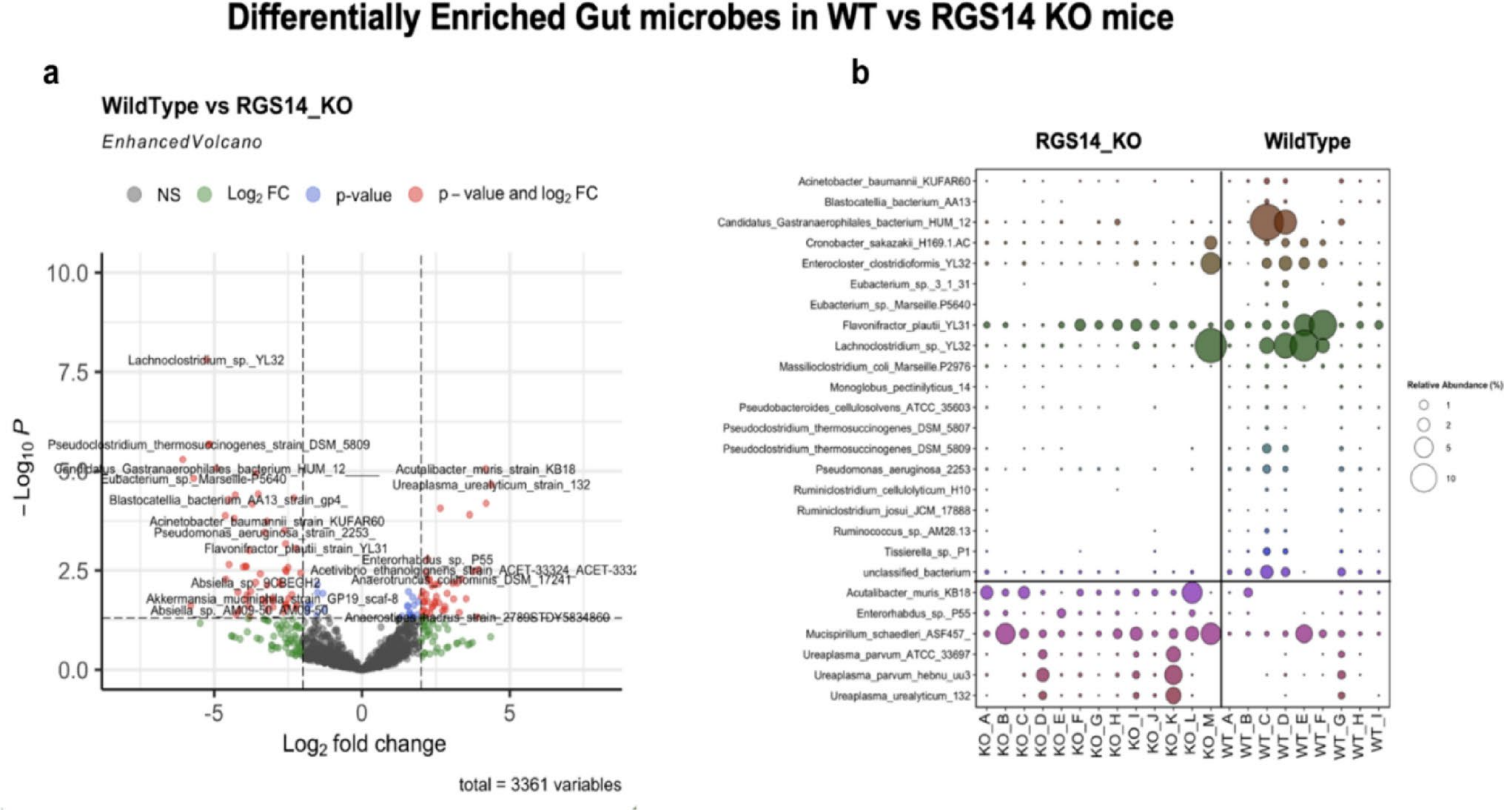
**A** Volcano plot showing differentially enriched and depleted rRNA operon reads matching bacteria at the strain level from the rRNA operon database (list provided in Supplementary Table 2). The vertical axis (Y-axis) corresponds to the mean expression value of log 10 (p-value) and the horizontal axis (X-axis) displays the log 2-fold change value. Positive x-values represent RGS14 KO and negative x- values represent WT littermates. **B** Relative abundance bubble plot identifying significantly enriched and depleted rRNA reads matching bacterial strains in RGS14 KO and WT littermate mice

Differential abundance analysis via DESeq2 was used to identify and visualize significantly enriched or depleted taxa between the two groups of mice at the strain level (Fig. 5a and b). rRNA operon reads, classified as 6 bacterial strains (*Acutalibacter muris* KB18, *Enterorhabdus sp.* P55, *Mucispirillum schaedleri* ASF457, *Ureaplasma parvum* ATCC 33697, *Ureaplasma parvum* hebnu uu3, and *Ureaplasma urealyticum* 132) were significantly enriched in RGS14 KO mice (Fig. 5a) while rRNA reads matching 20 strains in the rRNA operon database were depleted in RGS14 KO mice compared with their WT littermates (Suppl. Table 2).

#### Strain level analysis of additional taxa in RGS14 KO mice

**Fig. 6:**
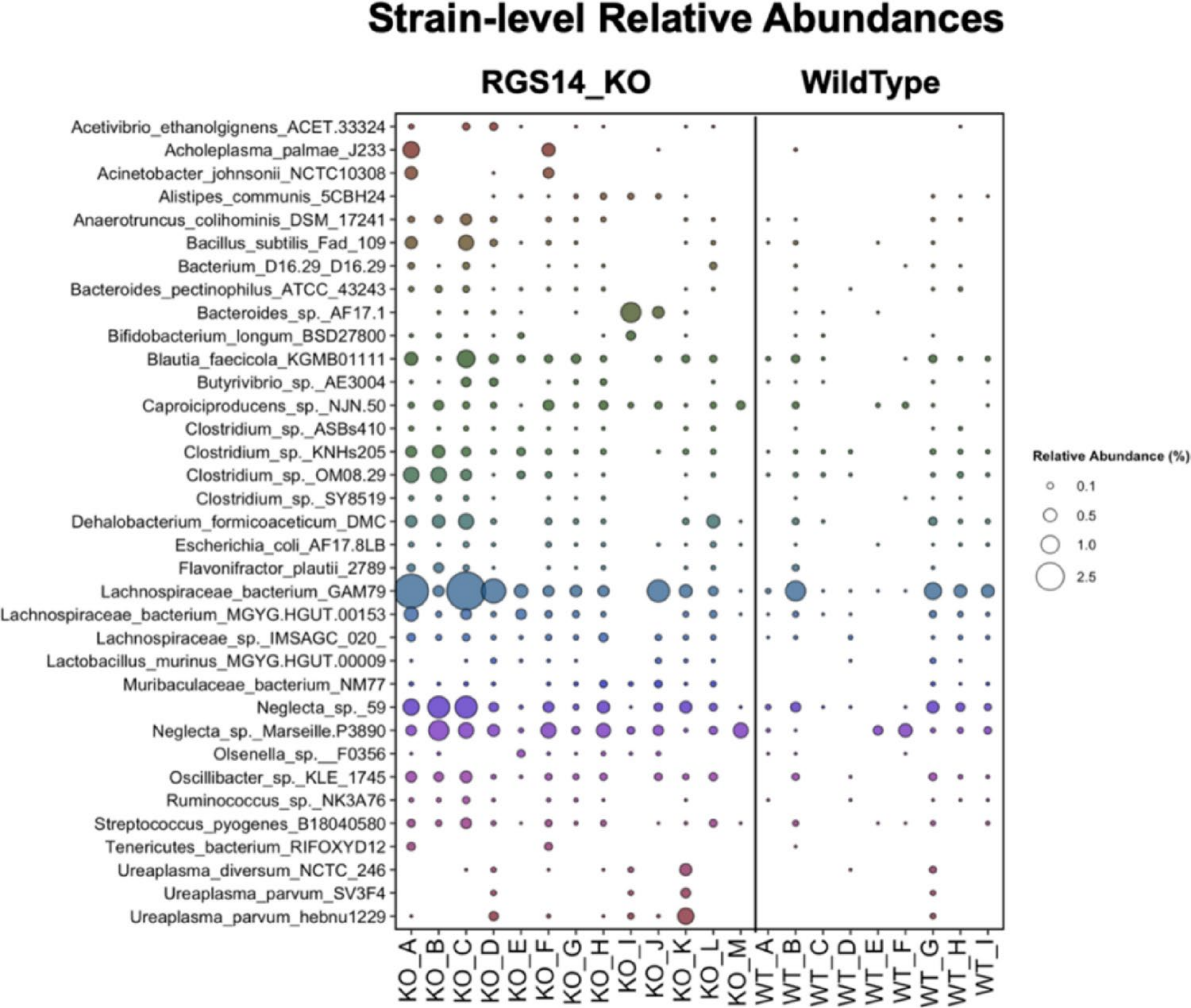
Relative abundance bubble plot of rRNA operon reads matching bacterial strains from the rRNA operon database in RGS14 KO vs. WT littermates which did not exceed the false discovery rate

There were rRNA operon reads matching over 70 additional bacterial strains which exhibited at least 4-fold enrichment in RGS14 KO mice. However, these taxa did not exceed the false discovery rate of 0.05 in the DESeq2 analysis. Of these, 38 taxa had raw reads < 50 across all RGS14 KO mice and are not considered here. While the remaining taxa were clearly more prevalent in RGS14 KO mice (Fig. 6), the differences in relative abundance were less stark than observed for the *Acutalibacter*, *Enterorhabdus*, *Mucispirillum*, and *Ureaplasma* strains (Fig. 5b). For example, rRNA reads matching two *Ureaplasma parvum* strains, *Ureaplasma diversum* NCTC 246, *Acholeplasma palmae* J233, and *Acinetobacter johnsonii* NCTC 10,308 were only observed in a few individual RGS14 KO mice and were either not detected in WT littermates or only observed in one WT individual. Other taxa were observed at slightly higher relative abundances in most RGS14 KO mice and sporadically in WT littermates. Finally, rRNA operon reads associated with some *Clostridium* sp. (ASBs410 and KNHs205), *Lachnospiraceae-associated bacterium* (GAM79), or *Neglecta* sp. (59 and Marseille P3890) had much higher relative abundances in select RGS14 KO mice compared with WT littermates.

#### Antibiotics impact gut microbiota of RGS14 KO and their WT littermates

**Fig. 7:**
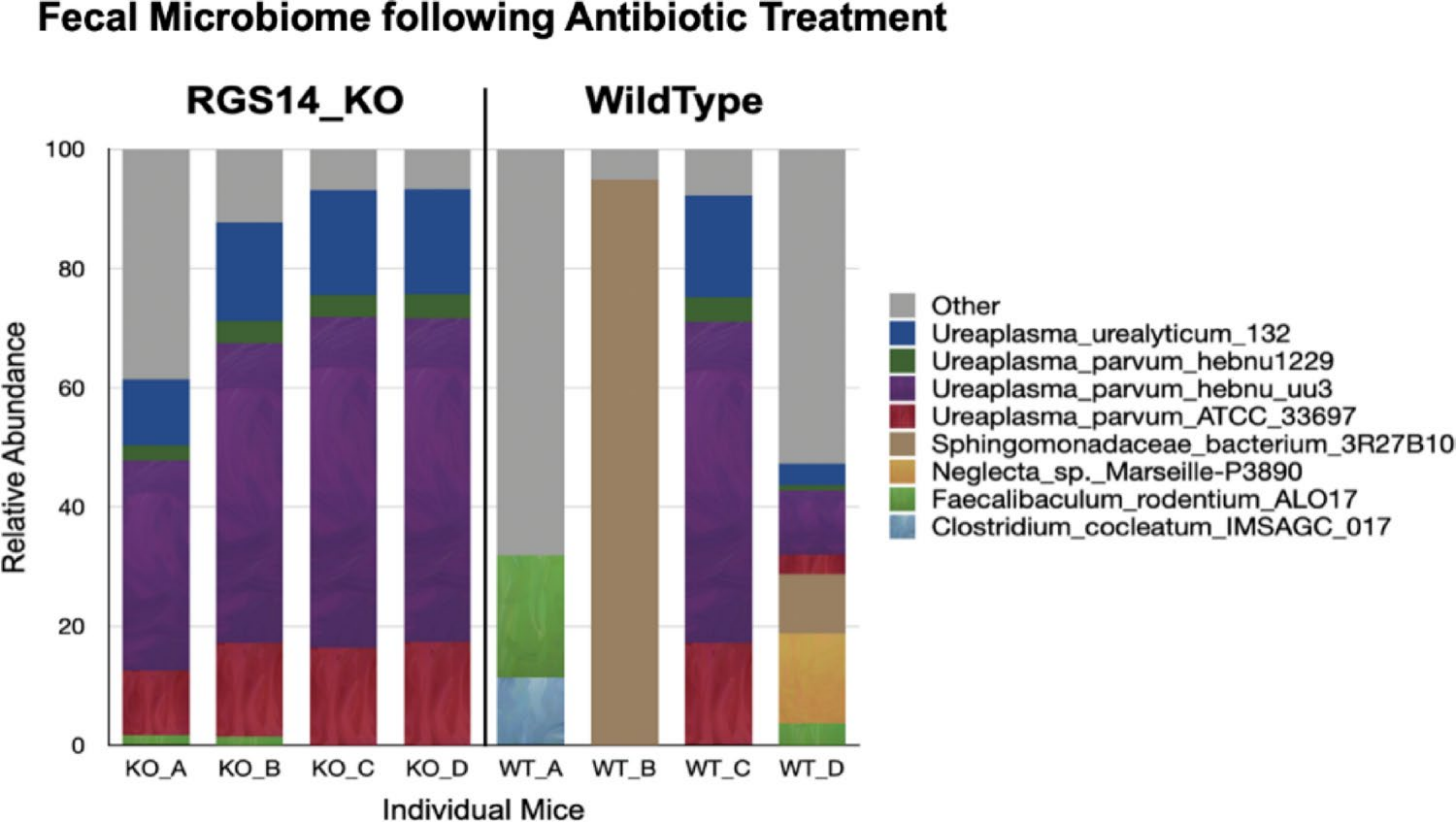
The total number of bacterial taxa was reduced from 3,360 before ABX to less than 1,400 after ABX

A total of 116,863 rRNA operon reads from antibiotic-treated RGS14 KO and WT mice were classified against the ribosomal RNA operon database. Close to 1,400 taxa were detected in the fecal samples following ABX compared with 3,360 taxa in the untreated sample set, although the overall amount of PCR amplicon was greatly reduced. Most taxa identified the RGS14 KO mice following ABX matched closely to *Ureaplasma* sp. (87% relative abundance; Fig. 7), while WT littermates only harbored 34% *Ureaplasma* sp.

## Discussion

The prevalence of metabolic diseases, such as obesity and type II diabetes increases with age (McKee and Morley 2000). The gut microbiome has been linked to healthy aging and longevity though production of metabolites such as BCAAs and short-chain fatty acids, which contribute to mitochondrial health (Badal et al. 2020; Bana and Cabreiro 2019). BAT may be a therapeutic target for anti-obesity and anti-diabetes due to its role in thermogenic regulation and energy homeostasis (Xiao and Kang 2020). Conventional and GF mouse studies support the role of the gut microbiome in the metabolic activity of BAT (Mestdagh et al. 2012). Additionally, conventionalization of GF mice with the cecal microbiota of normal mice increases body fat and insulin resistance within 14 days (Backhed et al. 2004). Clearly, the gut microbiome plays an important role in both normal physiology and human diseases through metabolite production (Cani 2013; Karlsson et al. 2013; Qin et al. 2012; Ridaura et al. 2013; Turnbaugh et al. 2006, 2009; Krautkramer et al. 2021), which may interact with metabolic tissues such as BAT.

Exercise also supports healthy aging, improves body composition and insulin sensitivity, and enriches gut bacteria associated with the production of beneficial metabolites (Eckstrom et al. 2020; Borghouts and Keizer 2000; Campbell and Wisniewski 2017; Campbell et al. 2016). We previously demonstrated that the RGS14 KO mouse outperforms its WT littermates in total distance run and work to exhaustion, and this exercise capacity can be conferred via surgical transplantation of BAT from RGS14 KO mice into their WT littermates (Vatner et al. 2023). Prior to ABX, RGS14 KO mice significantly outrun their WT littermates; following ablation of the gut microbiota by administering ABX for one week, the RGS14 KO mouse no longer significantly outperformed its WT littermates (Fig. 2). We also demonstrated a phenotypic weakness in critical power production between RGS14KO mice and their WT littermates (Table 1), which may be influenced by the gut microbiota. These interactions between genotype and gut microbiota may partially explain the observed variability in sustained metabolic output. These outcomes are consistent with the findings from our group and others demonstrating the importance of the gut microbiota for exercise performance and capacity (Dowden et al. 2023; Hsu et al. 2015). Additionally, genetic background and microbiome status may influence the physiological boundary separating sustainable from non-sustainable exercise intensity, where phenotypic weakness potentially represents a dominant constraint across groups. Thus, analyzing performance outcomes relative to each individual will allow for further insights into the effects of gut microbiota disruption on exercise outcomes.

Previous work from our lab using the RGS14 KO mouse model established a higher mitochondrial number and mitochondrial cristae density in SKM (Vatner et al. 2023). Untreated RGS14 KO mice presented with significantly increased quadriceps citrate synthase and NO activity compared to WT littermates (Fig. 3). Citrate synthase is a marker of mitochondrial density (Vigelso et al. 2014) and NO contributes to many aspects of skeletal muscle physiology, such as blood flow and contraction (Reid 1998). Following ABX, RGS14 KO mice and their WT littermates exhibited similar levels of citrate synthase and NO activity. In untreated animals, 15 metabolites were identified as significantly increased in RGS14 KO SKM compared to WT littermates (Table 4), which were associated with the purine metabolism pathway (Suppl. Figure 4). Following ABX, these significances were no longer present. Both inosine and hypoxanthine, which were significantly increased in RGS14 KO SKM, are important to purine metabolism specifically in SKM (Zielinski et al. 2019). Additionally, WT littermate mice presented with significantly increased AMP compared to RGS14 KO mice. When energy needs increase, the attached phosphate group(s) of adenosine triphosphate can be cleaved to generate adenosine diphosphate or AMP (Dunn and Grider 2025). Regulation of purine synthesis and degradation is essential to maintain adenosine triphosphate levels in SKM (Brault and Terjung 2001). Since the RGS14 KO mouse consistently demonstrates an increased exercise capacity compared to its WT littermates, it is unsurprising that RGS14 KO SKM possesses increased basal metabolite levels associated with adenosine triphosphate, a critical molecule for exercise. Taken together, these findings suggest that RGS14 KO SKM and exercise performance is influenced by the gut microbiota, and reduction of total gut bacteria negatively affects exercise performance outcomes via decreases in mitochondrial and NO activity and disruption of purine metabolism.

The gut microbiome has been linked to healthy aging and longevity though maintenance of the intestinal barrier; disruption of the intestinal epithelial barrier promotes bacterial infiltration, activation of the immune response, and inflammation (Bana and Cabreiro 2019). Following ABX, WT littermate mice presented with a significant increase in circulating GM-CSF and IL-12 p40, which was not identified in RGS14 KO mice (Table 2). IL-12 p40 is the subunit of IL-12, a cytokine produced by phagocytic immune cells following infection or stimulation from bacterial by-products (Ethuin et al. 2003). GM-CSF induces differentiation and activation of dendritic cells and macrophages and increases following stimulation from inflammatory molecules such as ILs and tumor necrosis factor α (Francisco-Cruz et al. 2014). As such, both play an important role in signaling an immune response following injury or insult. An in vitro study examining the effects of antibiotics on GM-CSF secretion found that cephalosporins and cyclosporin inhibited GM-CSF secretion by T-lymphocytes. Vancomycin, an antibiotic used in combination with others in this study, was also examined but did not alter GM-CSF secretion (Lenhoff and Olofsson 1996). Considering antibiotics are not normally prescribed without an underlying diagnosis, our data provides valuable insights into possible changes to the circulating immune response in a healthy host following ABX. Further, RGS14 KO mice appear to be less affected systemically by ABX-induced changes, providing additional support that the RGS14 KO model supports healthful aging.

We hypothesized that RGS14 KO mice would have a distinct gut microbiome compared to WT littermates, and that this bacterial community contributed to the model of healthful aging and longevity (Vatner et al. 2018a). Although we did not detect significant differences in the gut microbiota at higher taxonomic levels (phylum, class, order, family; Suppl. Figures 5–8) between RGS14 KO and WT littermate mice, we did identify significantly different community structures at the genus (Suppl. Figure 10), species (Suppl. Figure 11), and strain (Fig. 4) levels. Our analysis identified the enrichment of distinct rRNA operon reads from bacterial strains related to *A. muris*, *U. urealyticum*, *U. parvum*, and *M. schaedleri* in RGS14 KO mice. *A. muris* is a strictly anaerobic bacterium within the family *Oscillospiraceae*. This family also contains the genus *Oscillospira*, which has been associated with leanness and is negatively associated with inflammatory bowel disease (Lagkouvardos et al. 2016; Gophna et al. 2017). Both *M. schaedleri* and *A. muris* co-occurred with the pathogenic *Ureaplasma* spp. in our RGS14 KO mouse model (Fig. 5b). *M. schaedleri* competes with *Salmonella* for anaerobic respiration substrates in the gut of mice, likely restricting infection and inhibiting virulence factor expression (Herp et al. 2019) and may protect RGS14 KO mice from opportunistic pathogens like ureaplasmas. Since the *Ureaplasma* spp. were enriched in the RGS14 KO mouse both before and after ABX, we do not expect this group to significantly contribute to the healthy aging phenotype represented by the RGS14 KO mouse model.

Healthy aging and longevity have been linked through gut microbiome metabolite production, such as BCAAs and the short-chain fatty acids acetate, propionate, and butyrate (Bana and Cabreiro 2019). *A. muris* possesses enzymes involved in the pentose phosphate pathway (Lagkouvardos et al. 2016), and some *Oscillospira* strains have been identified as gut symbionts that utilize glucuronate and display enrichment in various fasting animal models (Gophna et al. 2017; Kohl et al. 2014). Members of *Oscillibacter* possess homologs for the first (thiolase) and second enzymes (3-hydroxybutyryl-CoA dehydrogenase) in butyrate production (Gophna et al. 2017). Butyrate, a gut-derived short-chain fatty acid, contributes to the regulation of thermogenesis in BAT (Wang et al. 2020), highlighting the ability of the gut microbiota to influence distal tissues. Mitochondria in BAT can utilize BCAAs for thermogenesis and reduces systemic BCAA levels (Yoneshiro et al. 2019). In our study, isoleucine was significantly higher at baseline in the BAT of RGS14 KO mice when compared to WT littermates. BCAAs have anti-obesity properties, regulate satiety hormones, and promote muscle protein synthesis (Lynch and Adams 2014), supporting the healthy aging phenotype of the RGS14 KO mouse. The nucleoside guanosine was significantly increased in untreated RGS14 KO mice. The intracellular messenger 3′,5′-cyclic guanosine monophosphate is essential for normal adipogenic differentiation and BAT-mediated thermogenesis in mice (Reverte-Salisa et al. 2019). The BAT of RGS14 KO mice possessed significantly higher glycerophosphocholine compared to WT littermates before ABX. A by-product of phosphatidylcholine, glycerophosphocholine is a water-soluble choline compound that reduces age-related deterioration and improves beta-oxidation in older mice (Narukawa et al. 2020). Phosphatidylcholine is a primary component of all plasma lipoprotein classes (Cole et al. 2012), and choline metabolites produced by phosphatidylcholine modifications have been identified as important components in maintaining healthy cellular structure and proliferation (Ridgway 2013). These data identify the potential impact the gut microbiota and its associated metabolites may contribute to the RGS14 KO BAT activity. Additionally, there may be an increased resilience to damage at the cellular level, further supporting the improved health span and longevity observations from our previous analyses of the RGS14 KO mouse.

## Limitations

This study is not without limitations. While the GF mouse model is the highest standard of gut microbiota study design, these animals are costly to house and handle. To accommodate this financial constraint, antibiotic administration has been acknowledge as equal in utility to reduce overall gut microbial load, which we applied for these studies (Kennedy et al. 2018). The metabolomics data presented in this manuscript focuses on microbial and metabolomic differences between the RGS14 KO and their WT littermates. As such, we do not present a full list of all metabolites identified in both WT and RGS14 KO mice. Additional studies, including the use of tracers, are needed to fully determine the extent of gut microbiota-derived metabolites and their distal effects. The species/strain level detection of gut microbes was identified to its closest database match to provide strain level resolution for long-read MinION sequences with matches to the rRNA operon database (Kerkhof et al. 2022). The changes in mitochondrial activity reported here are assayed using kit-based testing to demonstrate disruption in enzymatic and measurable analyte concentrations; future studies will utilize direct measurements of mitochondrial respiration. Finally, we acknowledge that male mice were only used in this experiment, this is due to the female mice in these cohorts being used for other studies and budgetary constraints that did not allow for additional female to be tested.

## Conclusions

Our study links specific gut microbial species/strains and metabolites to the enhanced exercise and healthful aging phenotype of the RGS14 KO mouse. Our comparison of bacterial communities between RGS14 KO and WT littermates revealed differences in the gut bacteria at the species/strain-level. RGS14 KO mice, which have increased BAT, harbor bacteria with properties associated with protection against obesity and adverse health conditions, improved metabolism at the cellular level, and enhanced immune function. We also identified metabolites in the BAT and SKM of RGS14 KO mice that are actively involved in energy metabolism and cellular homeostasis. Administration of ABX reduces RGS14 KO exercise capacity, likely due to the shift in tissue-specific metabolites and decreases in beneficial gut microbiota such as *A. muris*. Future mechanistic studies designed to correlate active gut microbes with their associated metabolic activity would provide more information on how these specific bacteria are linked to the RGS14 KO phenotype.

## Supplementary Information

Below is the link to the electronic supplementary material.

Supplementary Material 1

## Abbreviations

ABX: Antibiotic treatment
AMP: Adenosine monophosphate
AMPK: Adenosine monophosphate kinase
BAT: Brown adipose tissue
BCAA: Branched-chain amino acid
GF: Germ free
GM-CSF: Granulocyte-macrophage colony stimulating factor
IL: Interleukin
NO: Nitric oxide
RGS14 KO: Regulator of G protein signaling 14 knockout
rRNA: Ribosomal RNA
SIRT1: Sirtuin-1
SIRT3: Sirtuin-3
SKM: Skeletal muscle
WAT: White adipose tissue
WT: Wild-type
