# Supplementary material for: Disruption of the gut microbiota in regulator of G protein signaling 14 knockout (RGS14 KO) mice alters the metabolome and reduces enhanced exercise capacity
Source: Eur J Appl Physiol. 2026 Mar 14;126(7):3859–77. doi: 10.1007/s00421-026-06191-z (PMC13380600; doi:10.1007/s00421-026-06191-z)
Supplement: Supplementary file 1 — Supplementary Material 1 [file 421_2026_6191_MOESM1_ESM.pptx]

## Slide 1
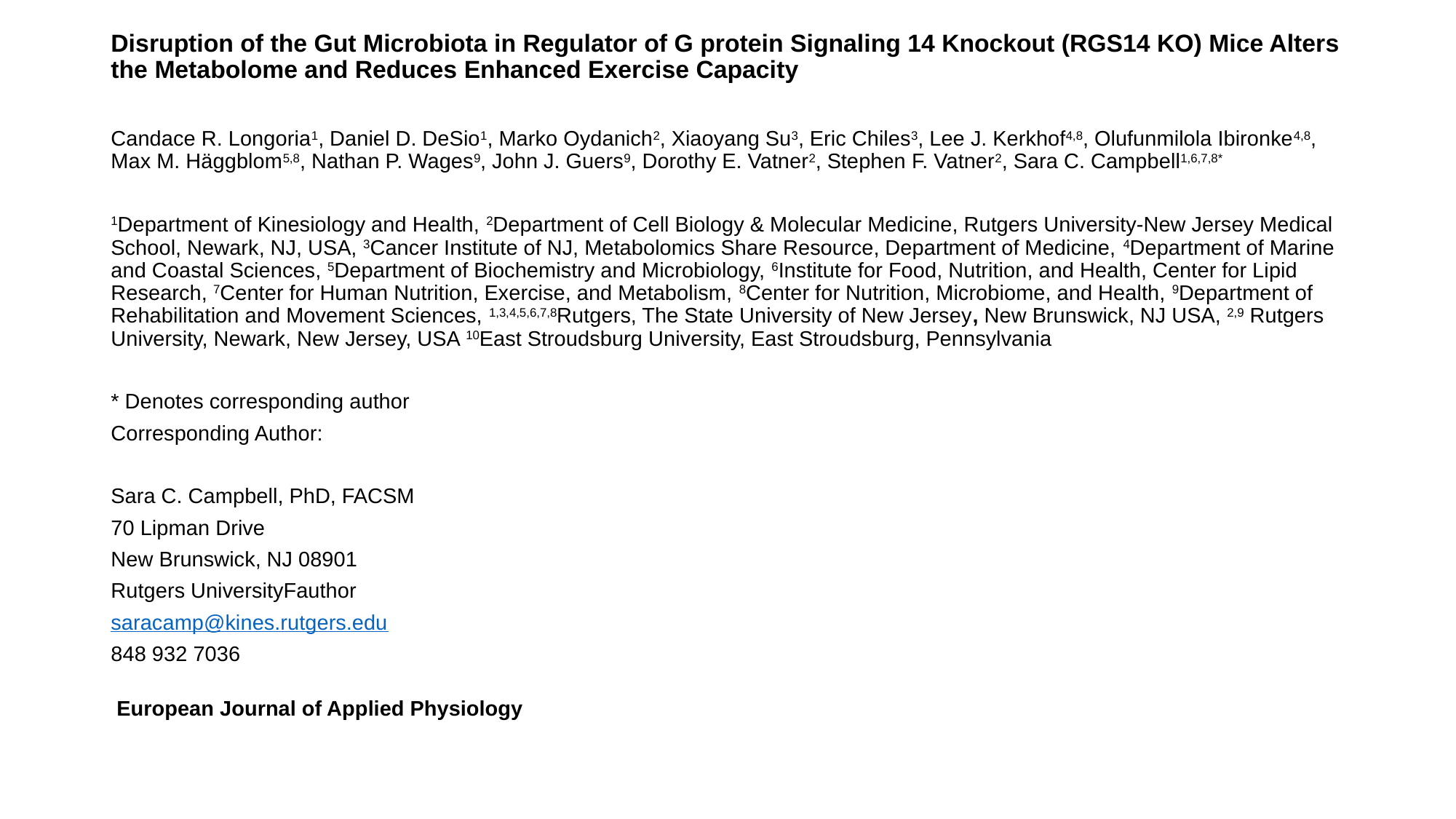

Disruption of the Gut Microbiota in Regulator of G protein Signaling 14 Knockout (RGS14 KO) Mice Alters the Metabolome and Reduces Enhanced Exercise Capacity
Candace R. Longoria1, Daniel D. DeSio1, Marko Oydanich2, Xiaoyang Su3, Eric Chiles3, Lee J. Kerkhof4,8, Olufunmilola Ibironke4,8, Max M. Häggblom5,8, Nathan P. Wages9, John J. Guers9, Dorothy E. Vatner2, Stephen F. Vatner2, Sara C. Campbell1,6,7,8*
1Department of Kinesiology and Health, 2Department of Cell Biology & Molecular Medicine, Rutgers University-New Jersey Medical School, Newark, NJ, USA, 3Cancer Institute of NJ, Metabolomics Share Resource, Department of Medicine, 4Department of Marine and Coastal Sciences, 5Department of Biochemistry and Microbiology, 6Institute for Food, Nutrition, and Health, Center for Lipid Research, 7Center for Human Nutrition, Exercise, and Metabolism, 8Center for Nutrition, Microbiome, and Health, 9Department of Rehabilitation and Movement Sciences, 1,3,4,5,6,7,8Rutgers, The State University of New Jersey, New Brunswick, NJ USA, 2,9 Rutgers University, Newark, New Jersey, USA 10East Stroudsburg University, East Stroudsburg, Pennsylvania
* Denotes corresponding author
Corresponding Author:
Sara C. Campbell, PhD, FACSM
70 Lipman Drive
New Brunswick, NJ 08901
Rutgers UniversityFauthor
saracamp@kines.rutgers.edu
848 932 7036
 European Journal of Applied Physiology

## Slide 2
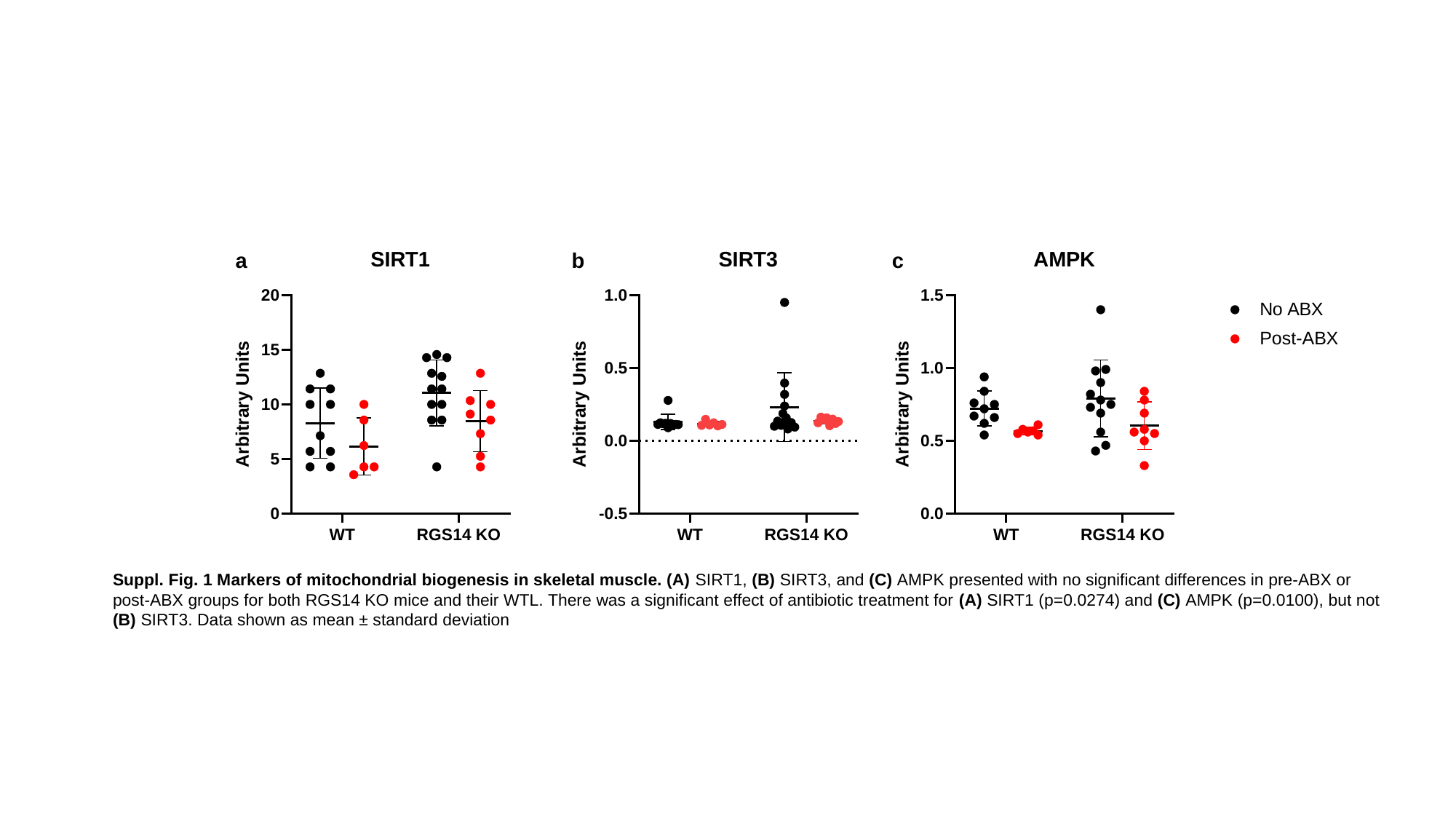

a
b
c
Suppl. Fig. 1 Markers of mitochondrial biogenesis in skeletal muscle. (A) SIRT1, (B) SIRT3, and (C) AMPK presented with no significant differences in pre-ABX or post-ABX groups for both RGS14 KO mice and their WTL. There was a significant effect of antibiotic treatment for (A) SIRT1 (p=0.0274) and (C) AMPK (p=0.0100), but not (B) SIRT3. Data shown as mean ± standard deviation

## Slide 3
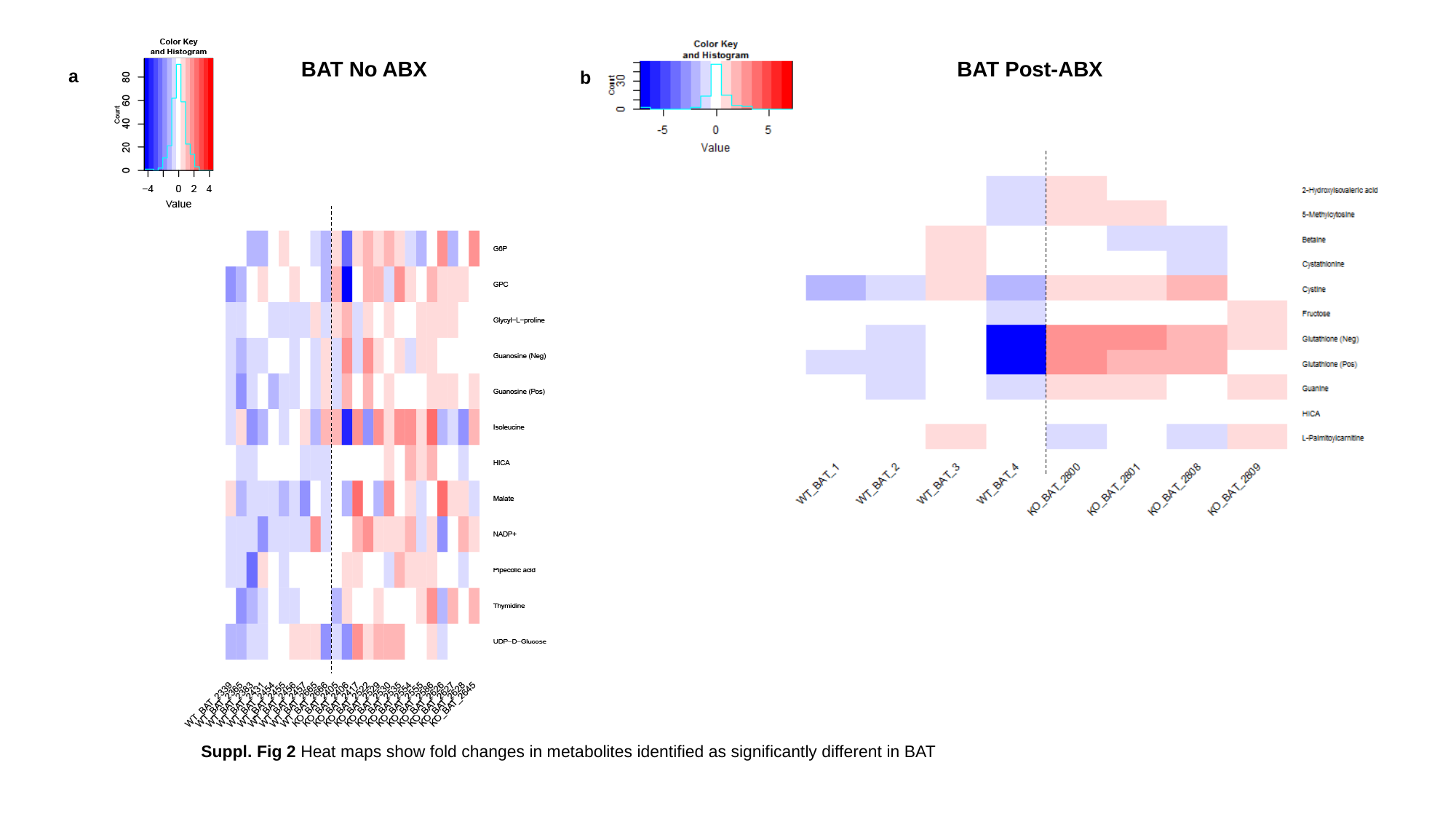

BAT No ABX
a
BAT Post-ABX
b
Suppl. Fig 2 Heat maps show fold changes in metabolites identified as significantly different in BAT

## Slide 4
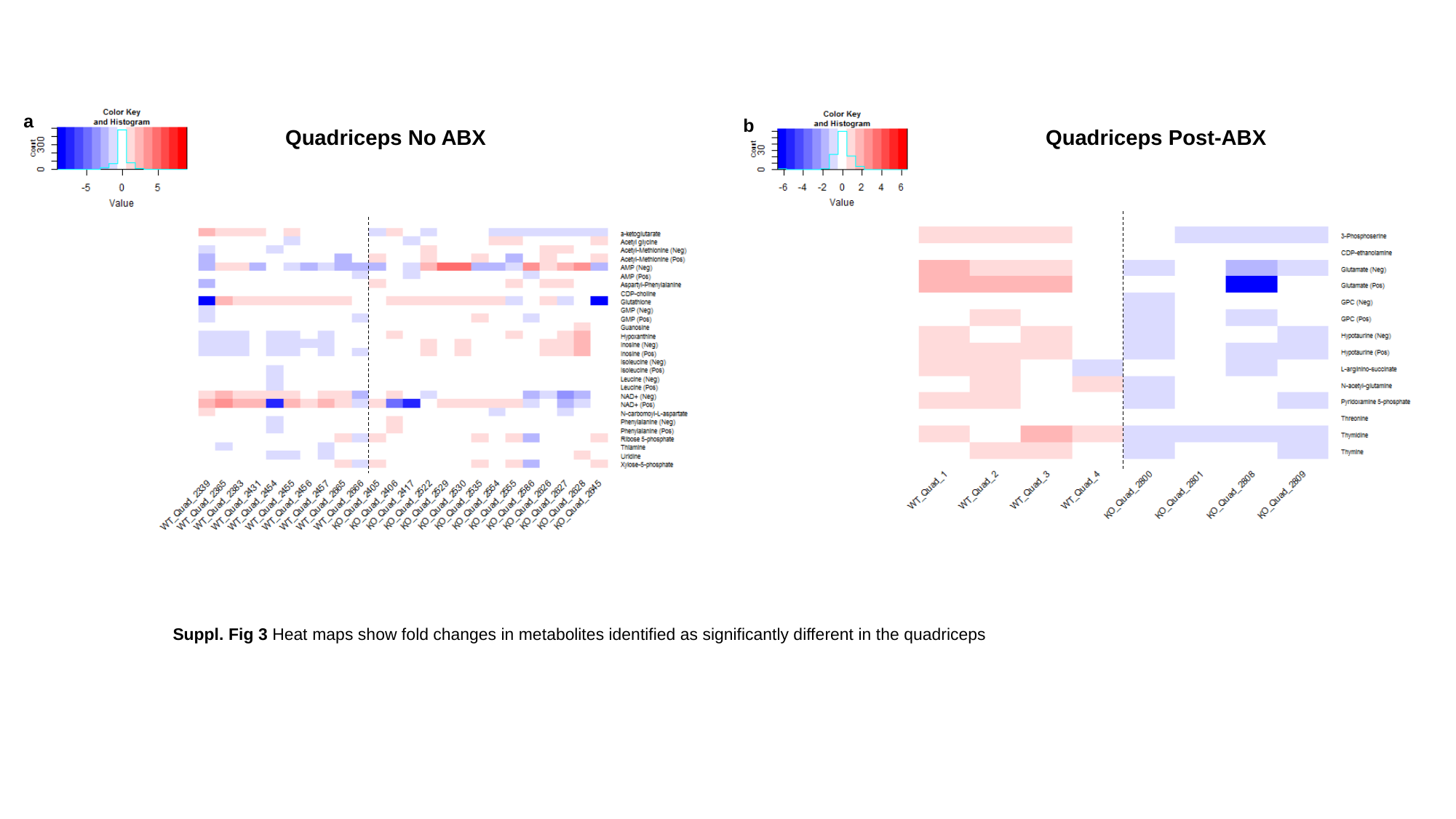

a
Quadriceps No ABX
b
Quadriceps Post-ABX
Suppl. Fig 3 Heat maps show fold changes in metabolites identified as significantly different in the quadriceps

## Slide 5
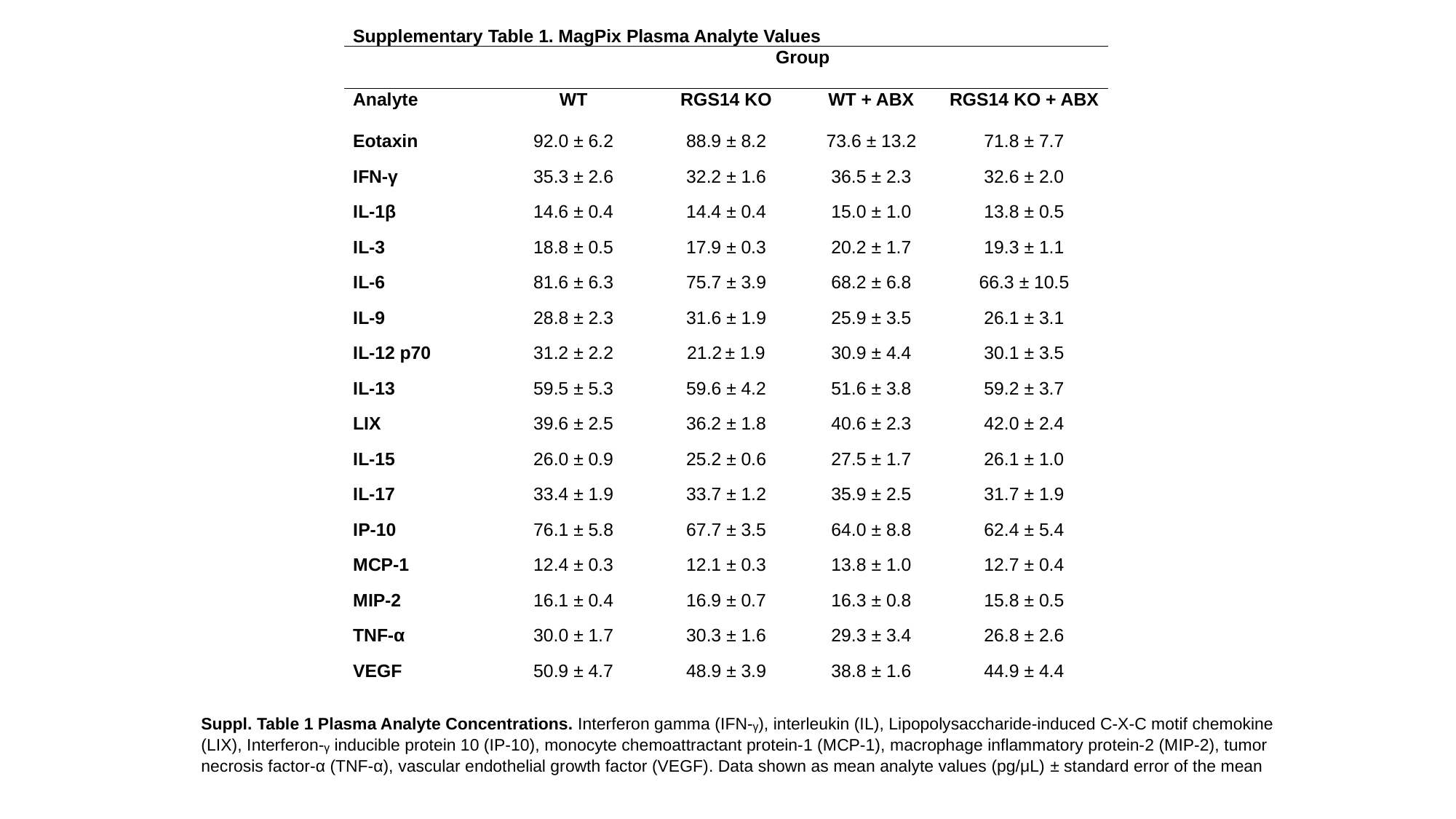

Suppl. Table 1 Plasma Analyte Concentrations. Interferon gamma (IFN-ᵧ), interleukin (IL), Lipopolysaccharide-induced C-X-C motif chemokine (LIX), Interferon-ᵧ inducible protein 10 (IP-10), monocyte chemoattractant protein-1 (MCP-1), macrophage inflammatory protein-2 (MIP-2), tumor necrosis factor-α (TNF-α), vascular endothelial growth factor (VEGF). Data shown as mean analyte values (pg/μL) ± standard error of the mean

## Slide 6
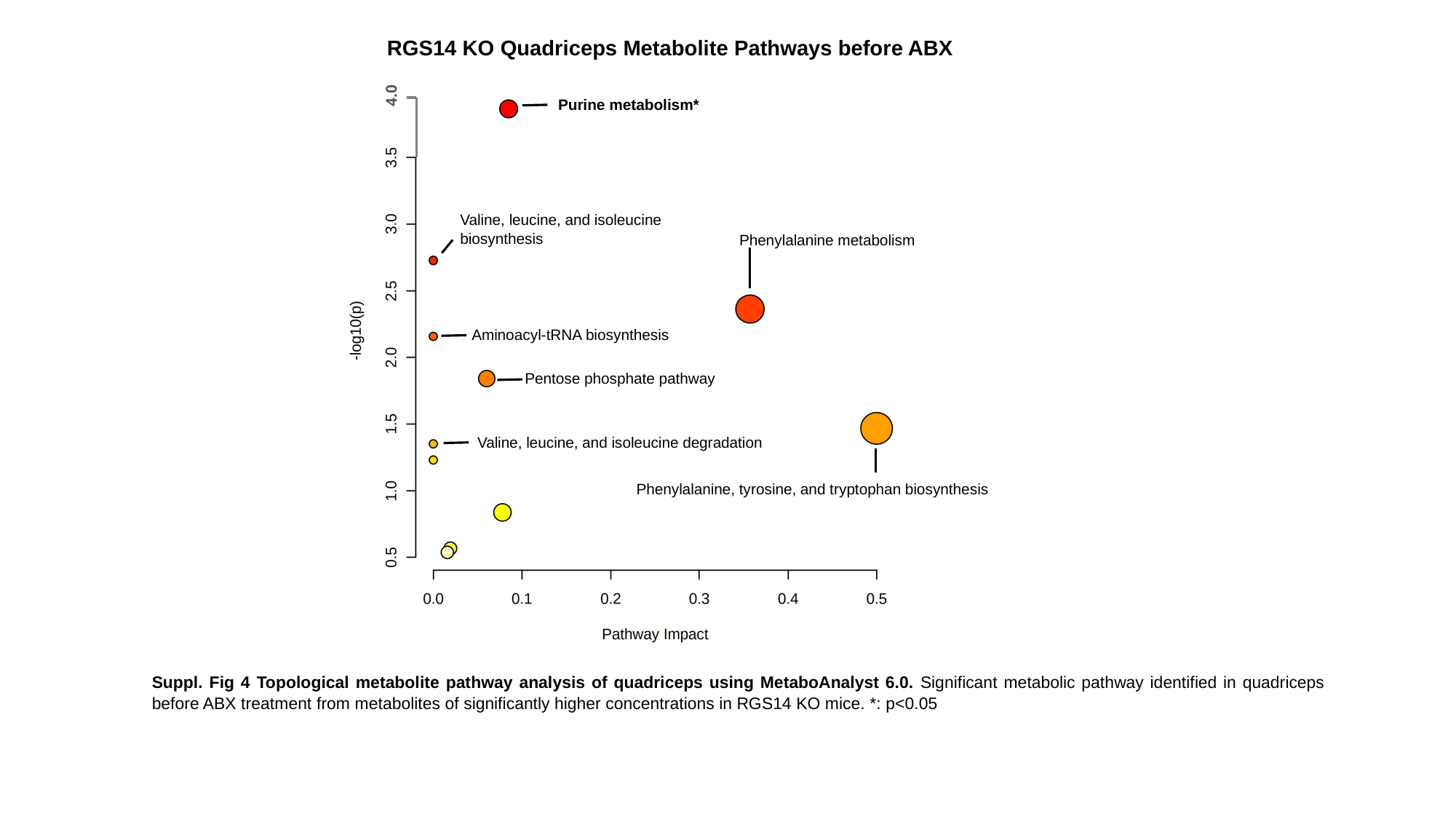

RGS14 KO Quadriceps Metabolite Pathways before ABX
4.0
Purine metabolism*
Valine, leucine, and isoleucine biosynthesis
Phenylalanine metabolism
Aminoacyl-tRNA biosynthesis
Valine, leucine, and isoleucine degradation
Phenylalanine, tyrosine, and tryptophan biosynthesis
Pentose phosphate pathway
Suppl. Fig 4 Topological metabolite pathway analysis of quadriceps using MetaboAnalyst 6.0. Significant metabolic pathway identified in quadriceps before ABX treatment from metabolites of significantly higher concentrations in RGS14 KO mice. *: p<0.05

## Slide 7
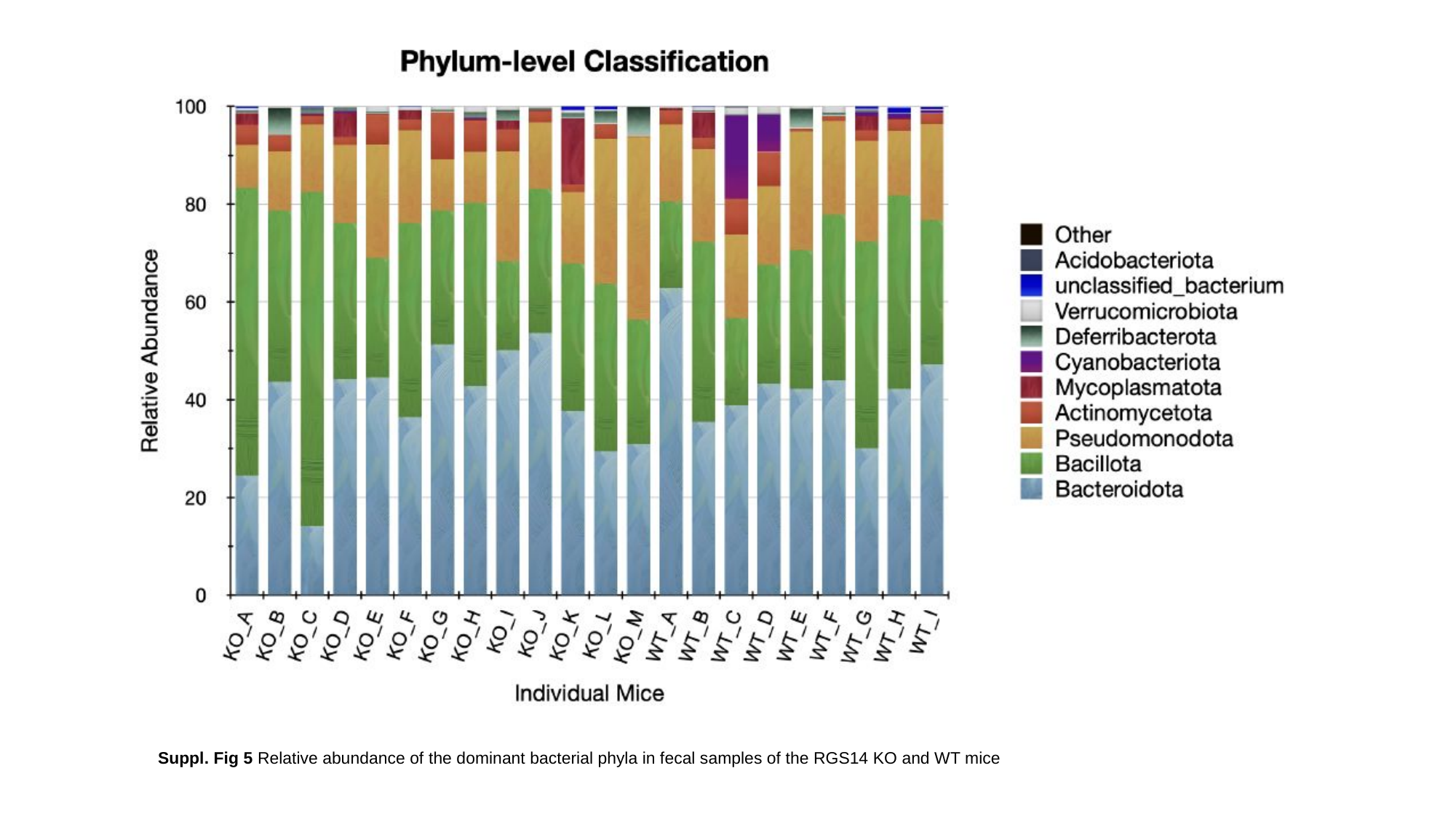

Suppl. Fig 5 Relative abundance of the dominant bacterial phyla in fecal samples of the RGS14 KO and WT mice

## Slide 8
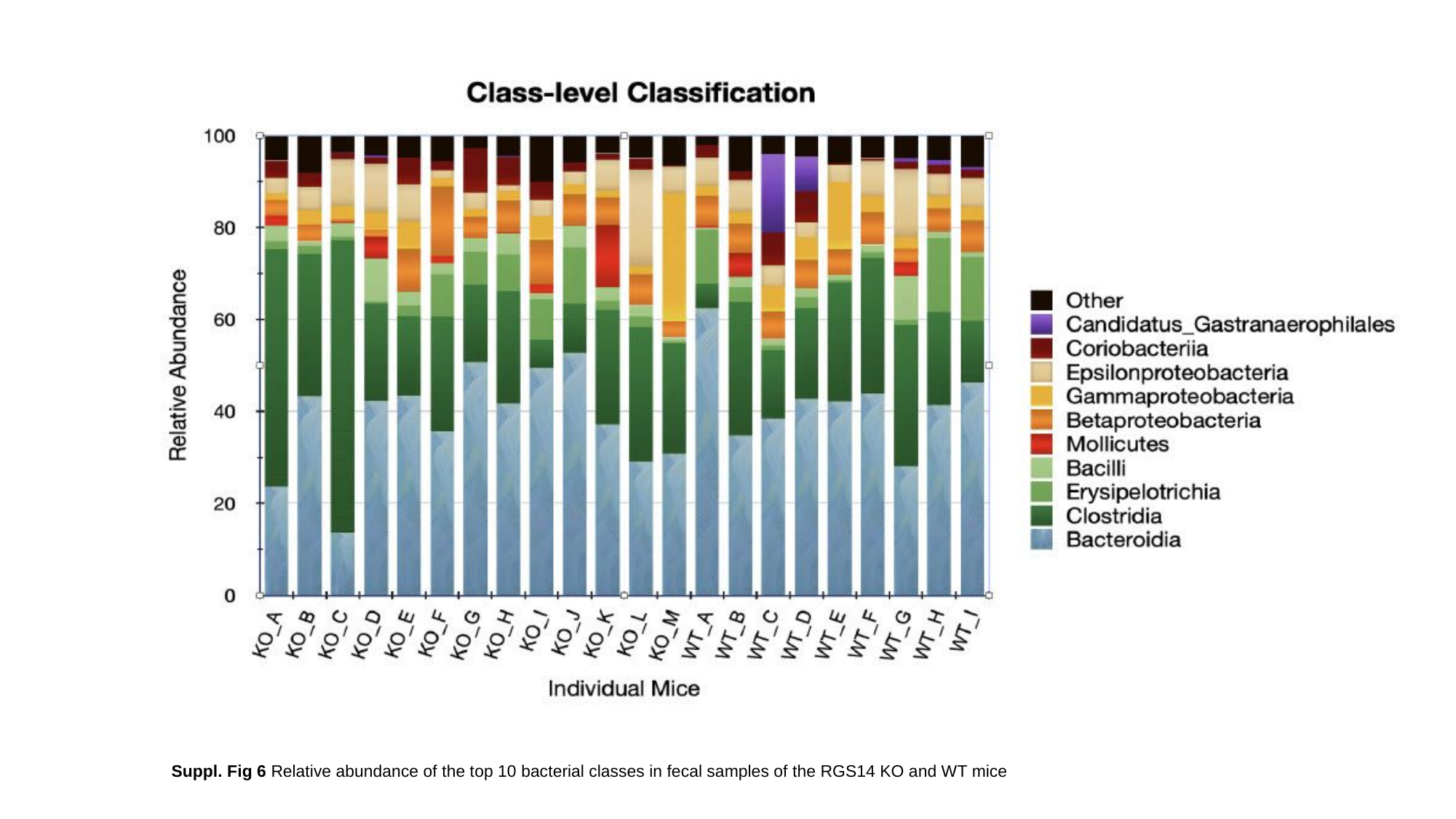

Suppl. Fig 6 Relative abundance of the top 10 bacterial classes in fecal samples of the RGS14 KO and WT mice

## Slide 9
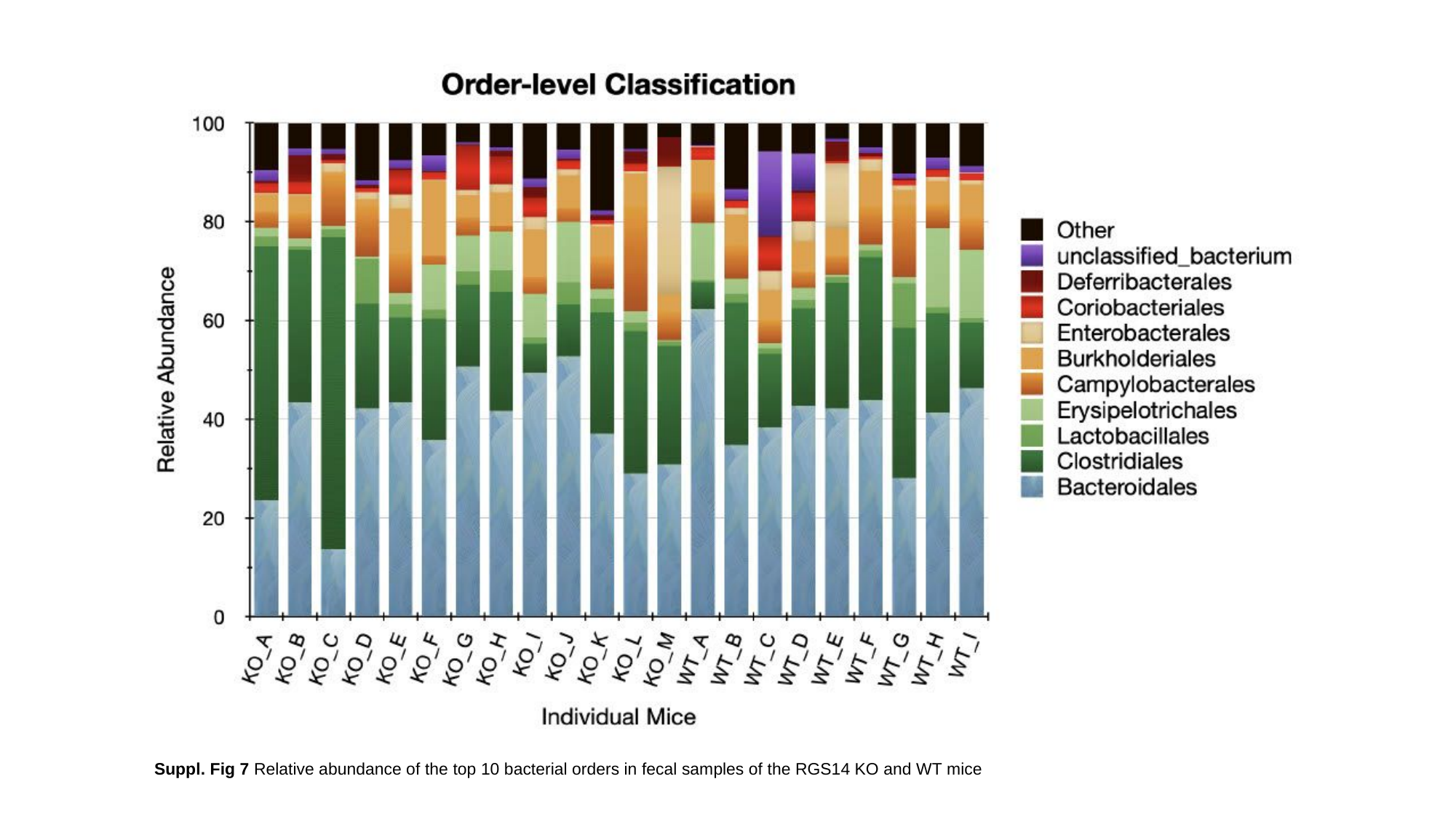

Suppl. Fig 7 Relative abundance of the top 10 bacterial orders in fecal samples of the RGS14 KO and WT mice

## Slide 10
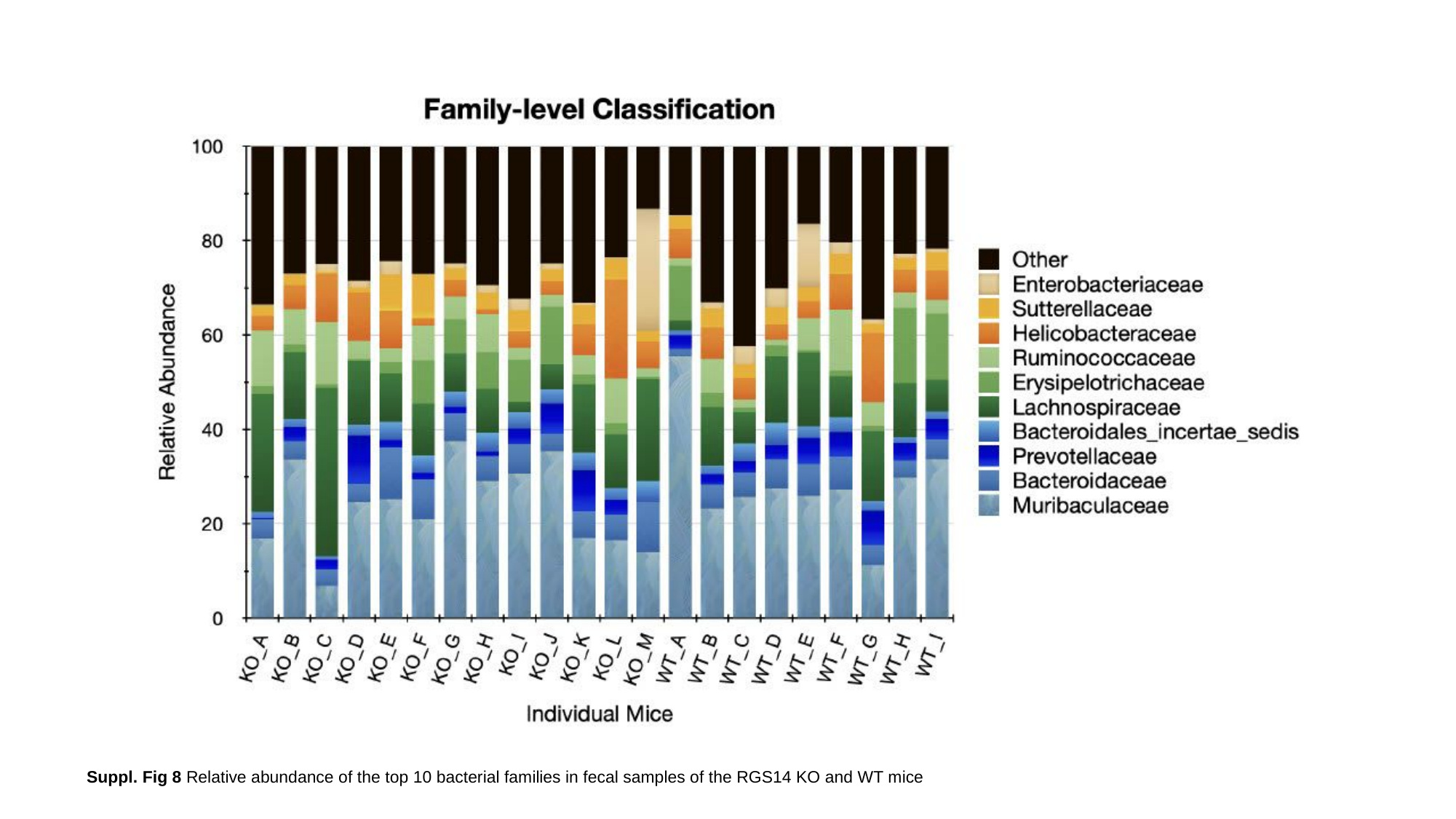

Suppl. Fig 8 Relative abundance of the top 10 bacterial families in fecal samples of the RGS14 KO and WT mice

## Slide 11
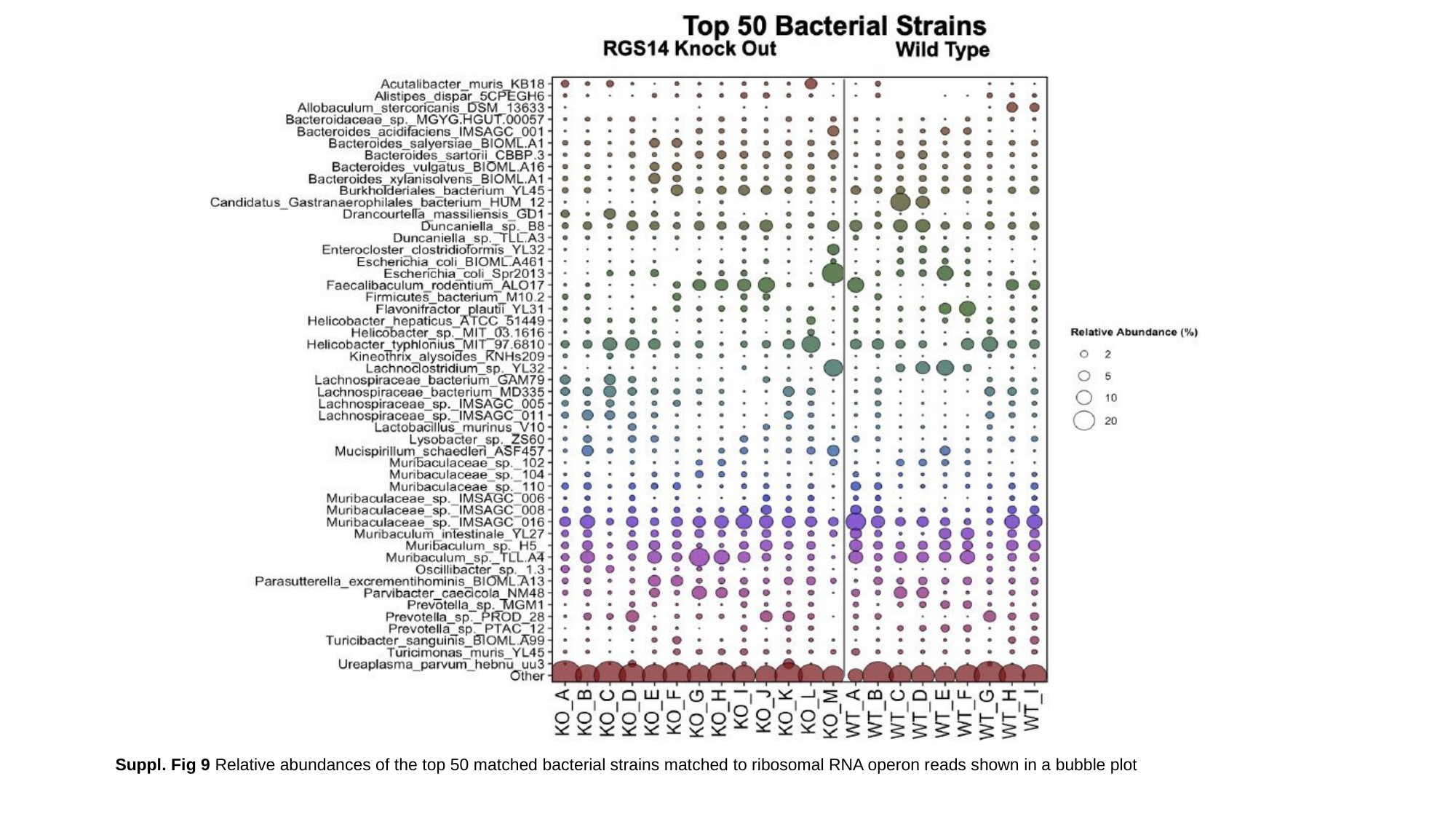

Suppl. Fig 9 Relative abundances of the top 50 matched bacterial strains matched to ribosomal RNA operon reads shown in a bubble plot

## Slide 12
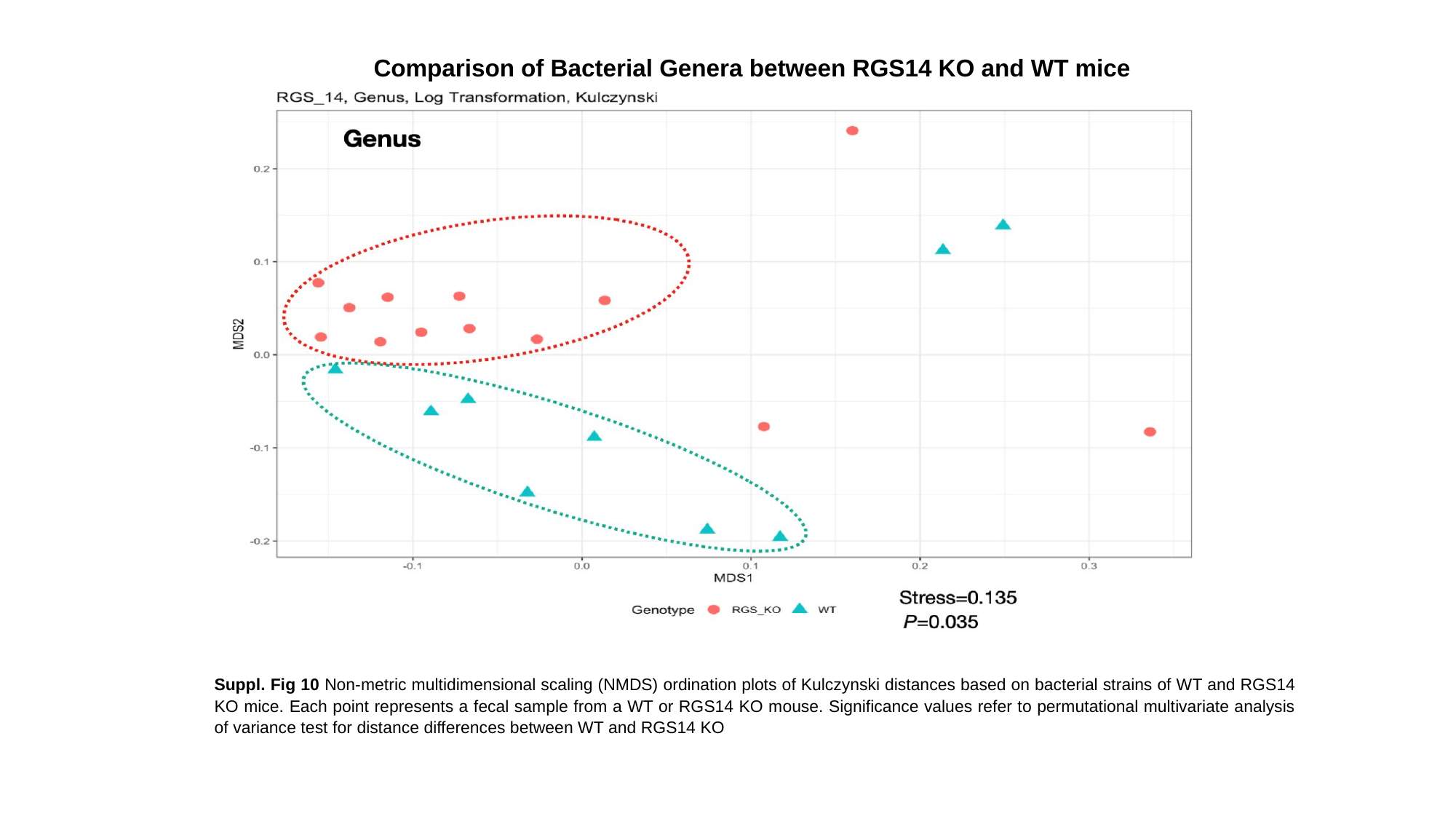

Comparison of Bacterial Genera between RGS14 KO and WT mice
Suppl. Fig 10 Non-metric multidimensional scaling (NMDS) ordination plots of Kulczynski distances based on bacterial strains of WT and RGS14 KO mice. Each point represents a fecal sample from a WT or RGS14 KO mouse. Significance values refer to permutational multivariate analysis of variance test for distance differences between WT and RGS14 KO

## Slide 13
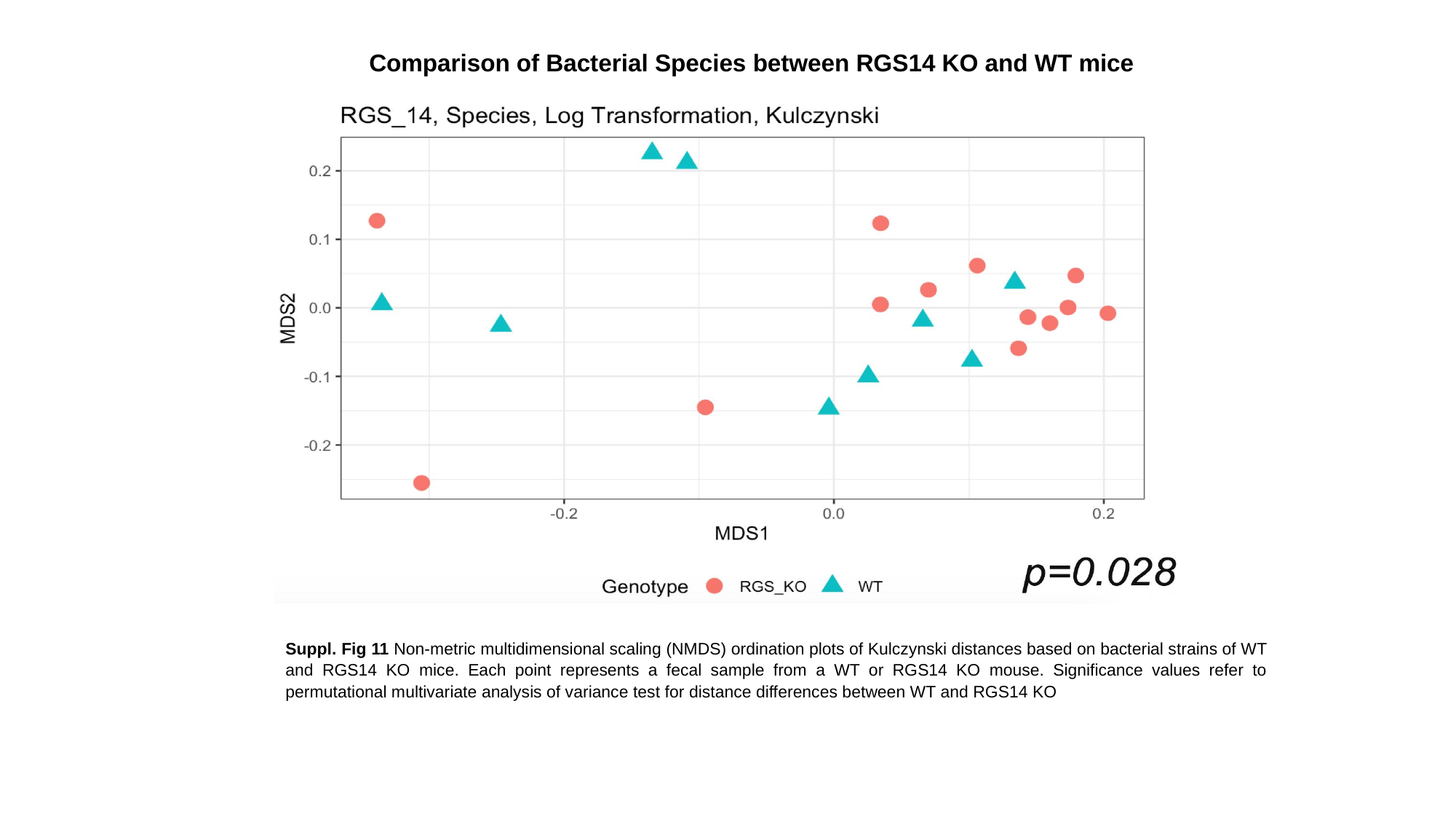

Comparison of Bacterial Species between RGS14 KO and WT mice
Suppl. Fig 11 Non-metric multidimensional scaling (NMDS) ordination plots of Kulczynski distances based on bacterial strains of WT and RGS14 KO mice. Each point represents a fecal sample from a WT or RGS14 KO mouse. Significance values refer to permutational multivariate analysis of variance test for distance differences between WT and RGS14 KO

## Slide 14
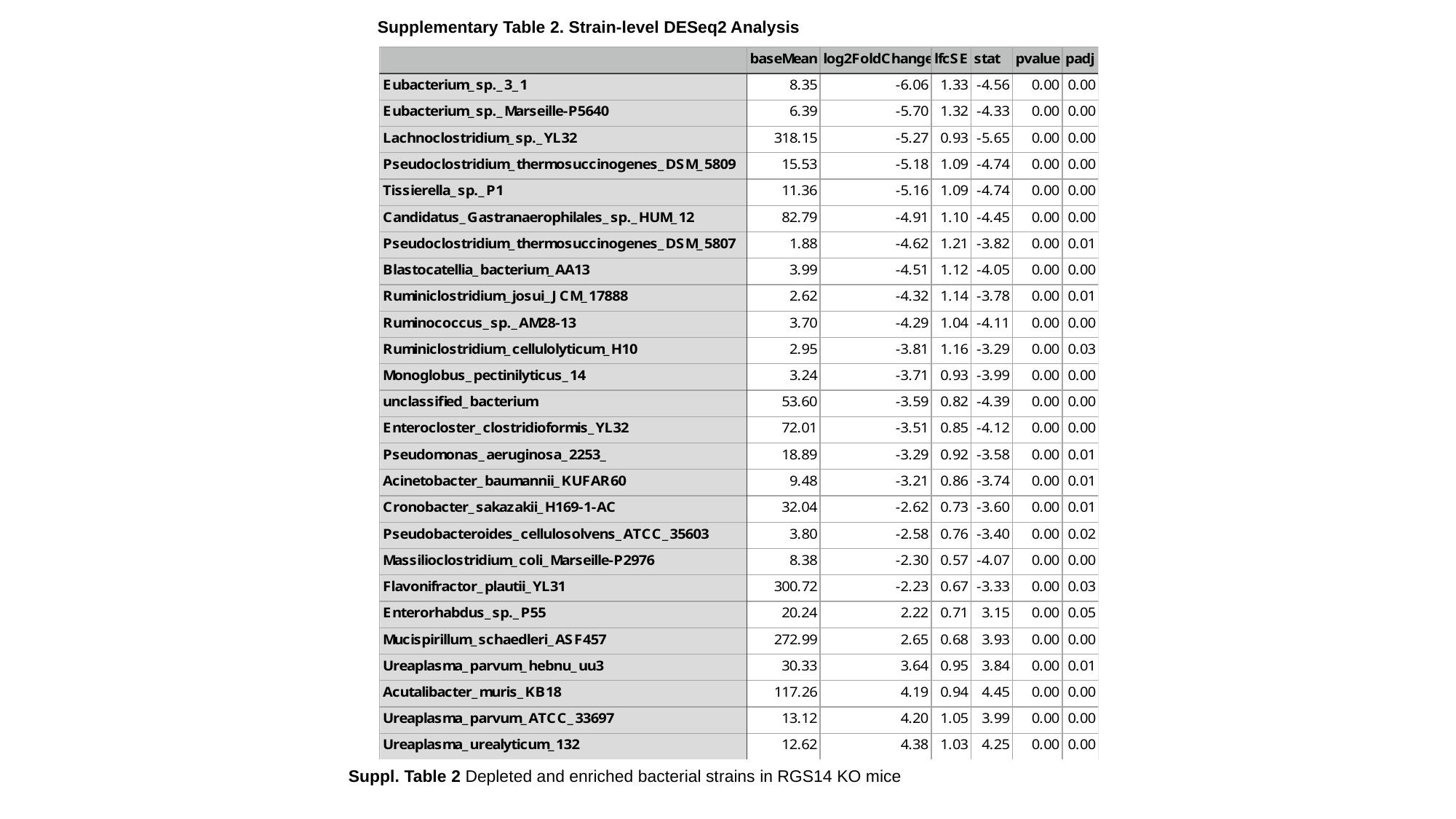

Supplementary Table 2. Strain-level DESeq2 Analysis
Suppl. Table 2 Depleted and enriched bacterial strains in RGS14 KO mice
